# Air Pollution Mixture and Folate Status Indicators among Pregnant Women in Canada: Maternal-Infant Research on Environmental Chemicals (MIREC) Study, 2008 to 2011

**DOI:** 10.1016/j.cdnut.2025.107617

**Published:** 2025-12-10

**Authors:** Tyler JS Smith, Hachem Saddiki, Joseph M Braun, Amanda J MacFarlane, Eric Lavigne, Markey Johnson, Ana Maria Siega-Riz, Raji Balasubramanian, Mandy Fisher, R Thomas Zoeller, Jillian Ashley-Martin, Tye E Arbuckle, Bruce P Lanphear, Youssef Oulhote

**Affiliations:** 1 Department of Environmental Medicine, Icahn School of Medicine at Mount Sinai, New York, NY, United States; 2 Department of Epidemiology, Brown University School of Public Health, Providence, RI, United States; 3 Nutrition Research Division, Health Canada, Ottawa, ON, Canada; 4 Department of Biology, Carleton University, Ottawa, ON, Canada; 5 Environmental Health Science and Research Bureau, Health Canada, Ottawa, ON, Canada; 6 School of Epidemiology and Public Health, University of Ottawa, ON, Canada; 7 Water and Air Quality Bureau, Health Canada, Ottawa, ON, Canada; 8 Department of Biostatistics & Epidemiology, University of Massachusetts at Amherst School of Public Health & Health Sciences, Amherst, MA, United States; 9 Department of Biology, University of Massachusetts at Amherst School of Public Health & Health Sciences, Amherst, MA, United States; 10 Faculty of Health Sciences, Simon Fraser University, Vancouver, BC, Canada

**Keywords:** air pollution, mixtures, folate status, prenatal nutrition, folic acid supplementation

## Abstract

**Background:**

Folic acid (FA) has been reported to modify associations between air pollutants and autism, but no study has investigated whether air pollutants are associated with maternal folate status.

**Objectives:**

We estimated associations between an air pollution mixture and maternal folate status among pregnant women in Canada.

**Methods:**

We enrolled pregnant women (*n* = 1983) in 10 cities in Canada from 2008 to 2011 in a prospective cohort. We estimated mean daily concentrations of nitrogen dioxide (NO_2_), ozone (O_3_), fine particulate matter (PM_2.5_), and sulfur dioxide (SO_2_) at maternal residences for 180 d preceding first and third trimester visits (ranges: 6–14 and 32–34 gestational weeks, respectively). At both visits, we measured plasma 5-methyltetrahydrofolate (5MTHF), unmetabolized FA (UMFA), and nonmethylated folate (NMF) concentrations and proportions (%5MTHF, %UMFA, %NMF). We used Bayesian weighted quantile sum regression to estimate expected differences in folate measures per 1-quartile difference in the air pollution mixture and weights for the mixture components. We stratified by fetal sex and FA supplementation.

**Results:**

Among complete cases in the first and third trimesters (*n* = 927 and 838, respectively), median (interquartile range) plasma total folate was similar between the first trimester [96.9 (78.6, 119.0) nmol/L] and the third trimester [99.6 (76.0, 135.0) nmol/L]. In the third but not the first trimester, the mixture was positively associated with plasma total folate [12.0 nmol/L per one-quartile increase, 95% confidence interval (CI): 1.5, 22.7 nmol/L], negatively associated with %5MTHF (–2.8, 95% CI: –5.1, –0.70), and positively associated with %UMFA (13.4, 95% CI: 4.4, 23.0). O_3_ was the primary component (weights ≥0.4). Associations were stronger for women with female fetuses.

**Conclusions:**

Air pollution was positively associated with total folate concentrations and UMFA proportions and negatively associated with 5MTHF proportions. Associations were strongest in the third trimester and among pregnant women carrying female fetuses. Air pollution may influence maternal folate status during pregnancy.

## Introduction

In recent years, epidemiologic studies have reported associations between ambient air pollution exposure in pregnancy and autism spectrum disorder or autistic traits in children [1]. It is unknown, however, whether the maternal folate system, which is critical to fetal neurodevelopment, mediates or modifies these associations [[2], [3], [4]].

Folic acid (FA) supplementation during periconception, which reduces the risk of neural tube defects (NTD) [[5], [6], [7]]—has been associated with reduced risk of autism spectrum disorder in children [[8], [9], [10], [11]]. To prevent NTD, FA supplementation of 400 μg/d is recommended to all women who are pregnant or could become pregnant [12,13]. Canada also mandates FA fortification of white wheat flour and other enriched grain products [14]. As a result, the prevalence of folate inadequacy among pregnant women in Canada is low (∼20%) with FA supplementation being the primary determinant of folate status [15,16].

Even in folate replete populations, however, interindividual variability in folate metabolism occurs. After ingestion, dietary folate and FA are metabolized in the liver to form the biologically active 5-methyltetrahydrofolate (5MTHF), which carries and activates one-carbon groups for cellular biological reactions. When FA intake exceeds hepatic metabolic capacity, unmetabolized folic acid (UMFA) enters systemic circulation [17]. Differences in folate metabolism, not just total folate, may be relevant to mediation or modification of associations between air pollution and autism.

A critical piece of this puzzle is whether air pollution disrupts maternal folate metabolism during pregnancy. Previous studies have found negative associations between other toxic chemicals and folate concentrations. Among pregnant women in Ottawa, Canada, smoking was associated with 23% and 15% lower serum and red blood cell (RBC) folate, respectively [18]. Among nonpregnant adults, smoking was associated with 20% and 32% lower plasma and RBC folate and 12% lower 5MTHF as a proportion of total folate measured in buccal mucosal cells [19]. Lead has also been associated with reduced total folate among highly exposed adult workers [20]. Perfluorinated and polyfluorinated substances, metals, and polycyclic aromatic hydrocarbons were associated with lower RBC folate among United States adults [21], and paraben and phenol exposure was associated with lower RBC and serum folate among United States children and adolescents [22]. To our knowledge, associations between ambient air pollutants and maternal folate status have not been investigated in pregnant women. The aim of this paper was to estimate associations between an air pollution mixture, total folate, and folate vitamer concentrations and proportions among pregnant women in the first and third trimesters in Canada.

## Methods

### Study design and sample

The Maternal-Infant Research on Environmental Chemicals (MIREC) Study is a prospective pregnancy and birth cohort designed to assess the role of environmental chemicals in the health of pregnant women and their children in Canada. Detailed enrollment and procedures have been described elsewhere [23]. Briefly, MIREC recruited pregnant women (*n* = 2001; 1983 with final consent) in the first trimester (6–<14 gestational weeks) at 11 hospitals in 10 Canadian cities from 2008 to 2011. Inclusion criteria included: ability to consent and to communicate in English or French, ≥18 y of age, <14 gestational weeks, willingness to provide a sample of cord blood, and intent to deliver at a local hospital. Exclusion criteria included: known fetal abnormalities or fetal chromosomal or major malformations in the current pregnancy, or a history of certain medical complications [23]. Here, we report estimates of prospective associations between air pollutant mixtures and folate status indicators among 927 women in the first trimester (6–<14 wk) and 838 women in third trimester (32–34 wk) who had singleton pregnancies and complete data. MIREC was approved by research ethics committees at Health Canada and all participating hospitals. All participants gave informed consent before enrollment.

### Air pollution mixture

We estimated daily concentrations of nitrogen dioxide (NO_2_), fine particulate matter (PM_2.5_), ozone (O_3_), and sulfur dioxide (SO_2_) at maternal residences. For each participant, we then calculated the mean daily concentration of each pollutant over the 180 d preceding first- and third-trimester study visits. The air pollution estimation methods have been described elsewhere [24,25]. Briefly, PM_2.5_ concentrations were derived from satellite estimates. Column aerosol optical depth (AOD) from multiple satellite retrievals and simulation were combined and related to near-surface PM_2.5_ using the spatially and temporally varying relationship between AOD and PM_2.5_ as simulated by a chemical transport model. Ground-based observations were then incorporated into these geophysically based estimates using a geographically weighted regression, providing an *R*^2^ of 0.70 with cross-validated ground-based observations [26]. NO_2_ concentrations were derived from a national land use regression (LUR) model developed using satellite NO_2_ estimates and geographic predictors scaled temporally with ground monitors from the National Air Pollution Surveillance (NAPS) network [24,25,27]. Sensitivity analyses were conducted by using the NO_2_ estimates from a national NO_2_ LUR model [27,28]. The national model had an *R*^2^ of 0.73 and a root mean square error of 2.9 parts per billion (ppb). O_3_ and SO_2_ concentrations were derived from NAPS observations alone. Maternal residences were the population-weighted centroids of the forward sortation areas (FSAs) (i.e., the first 3 letters of the postal code) in which participants lived during pregnancy, which were determined by questionnaire at the study visits. To reduce measurement error, participants living >30 km from a NAPS monitoring station, participants living in FSAs >20 km^2^, and participants missing >25% of daily air pollution concentration values were excluded from these analyses.

### Folate status indicators

At the first- and third-trimester visits, maternal whole blood was collected by venipuncture into vacutainer tubes containing EDTA [29]. Whole blood was centrifuged at 1500 × *g* for 15 min at 4°C to separate plasma, which was aliquoted and stored at –80°C until analysis. Liquid chromatography-tandem mass spectrometry (LC-MS/MS) was used to measure concentrations of 5 folate vitamers: 5MTHF, UMFA, tetrahydrofolate, 5,10-methenylTHF, and 5-formylTHF, as described elsewhere [29]. Nonmethylated folate (NMF) was calculated by summing concentrations of tetrahydrofolate, 5,10-methenylTHF, and 5-formylTHF. Total folate was calculated by summing 5MTHF, UMFA, and NMF. To calculate proportions, we divided 5MTHF, UMFA, and NMF by total folate (%5MTHF, %UMFA, and %NMF).

### Folic acid supplementation

In the first and early second trimesters, we surveyed participants about FA supplement use, as described elsewhere [2]. Briefly, at 16 wk of gestational age, we surveyed participants about FA supplementation in the past 30 d and 24 h. Additionally, at enrollment, we surveyed participants about “vitamin/mineral supplementation” in the past 3 mo. When available, the 30-d form was used. When the 30-d form was not returned or returned incomplete or empty, the 24-h and enrollment forms were used to estimate FA supplement intake. Surveys collected names and descriptions of supplements, product identification numbers (Drug Identification Number or Natural Product Number), amounts taken per time (e.g. number of pills per time), and frequencies of intake (e.g. number of times per day). We identified the FA content and recommended daily use from the Health Canada Licensed Natural Health Products Database or, if it was unavailable from the database, the manufacturer’s website. If a participant provided a brand but not the specific product, the mean FA content for all prenatal supplements from the manufacturer was assumed. If the FA content could not be determined from the Licensed Natural Health Products Database or the manufacturer’s website, the median FA content for all FA supplements identified in our sample (800 μg) was assumed. If the participant did not indicate the amount taken or the frequency of intake, the recommended daily intake from the manufacturer was assumed. FA intake was estimated as the product of FA content, amount taken, and frequency of intake (e.g. 400 μg/pill × 1 pill/time × 1 time/d = 400 μg/d). This estimate was categorized as <400 μg/d (not meeting the Health Canada recommendation for women who are pregnant or could become pregnant), 400 to 1000 μg/d (meeting the recommendation), or >1000 μg/d (exceeding the recommendation).

### Other variables

We selected adjustment variables a priori based on correlations with plasma total folate among pregnant women in MIREC [29]. The potential confounders were measures related to socioeconomic status, which may be related to levels of air pollution exposure: maternal education (high school or less, 2-y college or trade school, 4-y university degree, or graduate degree), household income (Canadian $0–50,000, 50,000–80,000, 80,000–100,000, or >100,000), household size (continuous in number of people), marital status (married/living with partner or not), maternal race/ethnicity (White or non–White), and maternal country of origin (Canada/United States or elsewhere). Other adjustment variables were measures unrelated to socioeconomic status that were included to increase precision: maternal age (continuous in years), parity (nulliparous, primiparous, or multiparous), FA supplementation in the 30 d before the first-trimester visit (<400, 400–1000, or >1000 μg/d), and a diet quality index (continuous, unitless), which was based on a food frequency questionnaire administered in the first trimester and measured alignment with the 2015 Dietary Guidelines for America [30]. In preliminary analyses, diet quality was positively associated with plasma total folate in the first and third trimesters among women taking <400 μg/d of FA (Supplemental Figure 1). Finally, we included fetal sex (male or female) to account for potential endocrine differences and study site to account for potential clustering.

### Statistical analysis

First, we calculated medians and interquartile ranges (IQRs) of plasma folate measures by participant characteristics. We used nonparametric Kruskal-Wallis tests to compare plasma folate measures by participant characteristics and between singleton pregnancies with complete plasma folate data and singleton pregnancies with complete plasma folate, air pollution, and confounder data; the latter were included in the full analysis. We also calculated the proportion of participants with plasma total folate <25.5 nmol/L, which is the cutoff for maximal NTD risk reduction [31]. Second, we used Bayesian weighted quantile sum regression (BWQS) to estimate *1*) expected differences in total folate, %5MTHF, %UMFA, %NMF, 5MTHF, UMFA, and NMF per one-quartile difference in the air pollution mixture over the 180 d preceding the first- or third-trimester study visit and *2*) weights reflecting what proportion of the expected difference is attributable to each mixture component (NO_2_, PM_2.5_, O_3_, and SO_2_). The Bayesian variant of weighted quantile sum regression does not require the analyst to specify the direction of the association (i.e. positive or negative) a priori [32]. For %5MTHF, %UMFA, and %NMF, we used a novel Dirichlet extension to BWQS [33] to model the proportions together as a multivariate outcome. This approach accommodated skewness and the compositional nature of the 3 proportions (i.e. for each participant, the proportions sum to one and a change in one value implies a change in at least one other value) [34,35]. Model parameters were estimated using a Markov chain Monte Carlo procedure with 2 independent chains, each with 30,000 iterations, including 15,000 burn-in iterations. Convergence was checked using trace, density, and autocorrelation plots [36]. We also stratified by fetal sex (male or female) and FA supplementation (<400, 400–1000, and >1000 μg/d). For Kruskal-Wallis and tests, alpha was set to 0.05. All statistical analyses were conducted in R [37].

## Results

Among 1983 pregnant women in MIREC, 1934 (98%) had singleton pregnancies (Supplemental Figure 2). Of these, 1802 (93%) and 1568 (82%) had complete plasma folate data in the first and third trimesters, respectively (Supplemental Figure 2). Of these, we included 927 (51%) and 838 (53%) pregnant women with complete plasma folate, air pollution, and confounder data in the first and third trimesters, respectively (Supplemental Figure 2). The primary type of missing data was air pollution, which accounted for 88% to 93% of incomplete cases among pregnant women with complete outcome data (Supplemental Figure 2). In the first trimester, we found small but statistically significant differences between complete and incomplete cases in %UMFA (2.8 compared with 2.6%, respectively; Kruskal-Wallis *P* = 0.05) and %NMF (2.2 compared with 2.1%, respectively; *P* = 0.01) (Supplemental Table 1). In the third trimester, no statistically significant differences (*P* > 0.05) were found for plasma folate measures between complete and incomplete cases (Supplemental Table 2).

In the first and third trimesters, median (IQR) NO_2_, PM_2.5_, O_3_, and SO_2_ were 22.5 (13.3, 30.2) and 21.3 (13.6, 28.7) ppb, 21.3 (18.2, 25.6) and 22.8 (19.4, 27.0) ppb, 10.2 (7.3, 13.4) and 9.9 (7.4, 13.9) μg/m^3^, and 1.5 (0.9, 1.9) and 1.3 (0.9, 1.7) ppb, respectively (Supplemental Table 3). Median (IQR) plasma total folate was similar between the first trimester [96.9 (78.6, 119.0) nmol/L] and the third trimester [99.6 (76.0, 135.0) nmol/L] (Table 1). At both visits, <1% of participants had plasma total folate <25.5 nmol/L, the cutoff associated with maximal NTD risk reduction. In both trimesters, the dominant vitamer was 5MTHF (medians >80%) followed by UMFA and NMF (medians <10%) (Supplemental Tables 1 and 2). In the first and/or third trimesters, we saw small but statistically significant differences in plasma total folate by age, parity, education, household income, household size, FA supplementation, and healthy eating index (Table 1).

**TABLE 1:** Median (interquartile range) plasma total folate in the first and third trimesters for singleton pregnancies with complete data by participant characteristics, MIREC study, Canada, 2008 to 2011.

Participant characteristic	First trimester		Third trimester	
	*n* (%)	Total folate (nmol/L)	*n* (%)	Total folate (nmol/L)
Overall	927 (100)	96.9 (78.6, 119.0)	838 (100)	99.6 (76.0, 135.0)
Age (y)		*P* < 0.0001		*P* < 0.0001
≤30	321 (35)	89.3 (73.0, 110.7)	292 (35)	90.8 (67.6, 119.9)
31–35	327 (35)	100.8 (82.6, 124.3)	303 (36)	104.9 (81.0, 144.7)
≥36	279 (30)	99.5 (81.6, 120.1)	243 (29)	105.8 (79.2, 147.6)
Parity		*P =* 0.02		*P* < 0.0001
0	411 (44)	99.3 (80.5, 124.9)	379 (45)	107.1 (84.8, 151.4)
1	368 (40)	95.5 (78.0, 114.4)	337 (40)	92.9 (71.9, 122.2)
≥2	148 (16)	94.8 (75.0, 110.2)	122 (15)	88.2 (56.8, 130.0)
Education		*P* = 0.04		*P* < 0.0001
High school diploma or less	219 (24)	96.9 (74.4, 124.1)	205 (24)	92.0 (65.3, 126.4)
College or trade school	283 (31)	97.1 (80.8, 115.5)	267 (32)	104.9 (81.4, 142.0)
University degree	69 (7)	88.4 (65.9, 110.6)	53 (6)	78.9 (47.6, 102.3)
Graduate degree	356 (38)	99.0 (80.8, 118.6)	313 (37)	101.1 (77.5, 139.4)
Household income		*P* = 0.03		*P* < 0.01
0–50	165 (18)	93.9 (67.2, 112.4)	151 (18)	92.0 (53.9, 130.3)
50–80	178 (19)	95.3 (78.3, 117.6)	179 (21)	93.5 (74.8, 125.5)
80–100	186 (20)	100.2 (80.7, 120.0)	150 (18)	105.5 (77.4, 166.4)
>100	398 (43)	97.2 (80.8, 120.8)	358 (43)	102.5 (80.4, 137.3)
Household size		*P* = 0.01		*P* < 0.0001
1	40 (4)	115.0 (94.3, 138.0)	40 (5)	111.6 (79.6, 149.9)
2	385 (42)	97.9 (79.4, 124.0)	354 (42)	106.0 (83.9, 151.1)
3	365 (39)	94.7 (78.1, 113.9)	330 (39)	93.2 (72.0, 123.6)
≥4	137 (15)	94.9 (78.0, 109.9)	114 (14)	90.1 (58.2, 118.6)
Marital status		*P* = 0.79		*P* = 0.31
Married or partnered	877 (95)	96.6 (78.7, 117.5)	792 (95)	99.6 (76.4, 134.8)
Not married	50 (5)	99.2 (64.7, 127.5)	46 (5)	99.0 (59.3, 133.3)
Race/ethnicity		*P* = 0.11		*P* = 0.28
White	737 (80)	97.3 (79.2, 119.7)	672 (80)	99.9 (76.4, 137.6)
NonWhite	190 (20)	94.5 (77.1, 113.1)	166 (20)	98.1 (72.6, 123.9)
Country of origin		*P* = 0.18		*P* = 0.06
Canada or United States	740 (80)	96.5 (78.2, 118.7)	674 (80)	98.2 (74.9, 131.7)
Elsewhere	187 (20)	97.7 (83.4, 119.3)	164 (20)	104.0 (81.2, 149.9)
Folic acid supplementation		*P* < 0.0001		*P* < 0.0001
<400	59 (6)	73.7 (57.0, 95.5)	55 (7)	66.0 (45.5, 101.8)
400–1000	623 (67)	96.5 (79.9, 114.7)	570 (68)	100.1 (76.8, 138.6)
>1000	245 (26)	103.1 (81.4, 127.5)	213 (25)	103.6 (82.2, 134.6)
Healthy eating index		*P* = 0.67		*P =* 0.02
Tertile 1	276 (30)	95.4 (75.7, 121.8)	239 (29)	94.5 (61.3, 130.5)
Tertile 2	306 (33)	97.1 (79.4, 114.9)	283 (34)	98.3 (77.1, 141.6)
Tertile 3	345 (37)	97.3 (81.6, 117.8)	316 (38)	103.7 (81.7, 135.2)
Fetal sex		*P* = 0.33		*P* = 0.56
Male	489 (53)	97.7 (79.8, 119.7)	453 (54)	100.5 (76.5, 136.5)
Female	438 (47)	95.6 (77.9, 117.2)	385 (46)	99.3 (75.5, 130.3)

Data are for singleton pregnancies with complete plasma folate, exposure, and confounder data in the Maternal-Infant Research on Environmental Chemicals (MIREC) Study. Plasma total folate was measured in the first trimester (6–<14 gestational wk) and third trimester (32–34 wk) using liquid chromatography with tandem mass spectrometry (LC-MS/MS). *P* values were calculated by Kruskal-Wallis tests.

**FIGURE 1:**
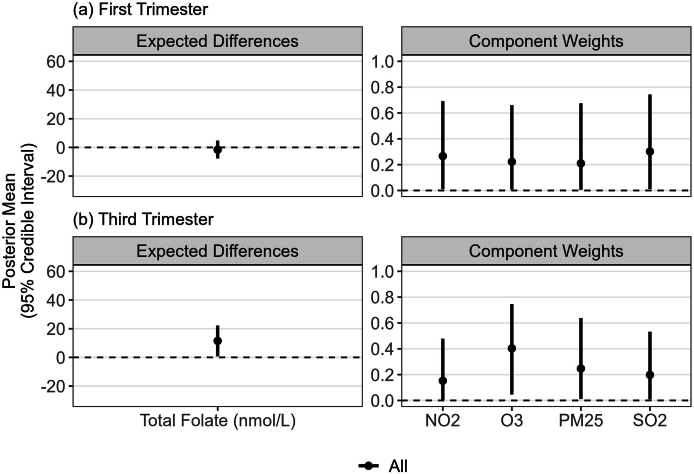
Mean (95% credible interval) posterior estimates of expected differences in plasma total folate per 1-quartile difference in air pollution mixture and component weights in the first and third trimesters among pregnant women in Canada, MIREC Study, Canada, 2008 to 2011. Parameters were estimated using Bayesian weighted quantile sum regression models with 2 independent chains, each with 30,000 iterations, including 15,000 burn-in iterations. Models were adjusted for maternal age, parity, education, household income, household size, marital status, race/ethnicity, country of origin, folic acid supplementation, healthy eating index, study site, and fetal sex. NO_2_, nitrogen dioxide; O_3_, ozone; PM_2.5_, particulate matter 2.5; SO_2_, sulfur dioxide.

**FIGURE 2:**
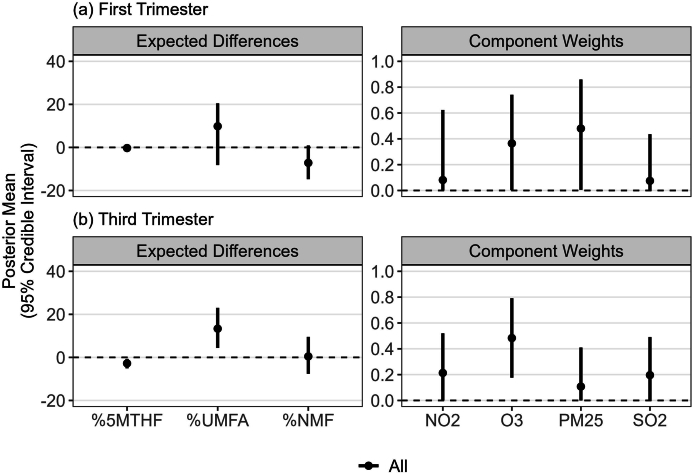
Mean (95% credible interval) posterior estimates of expected differences in folate vitamer proportions per 1-quartile difference in air pollution mixture and component weights in the first and third trimesters among pregnant women in Canada, MIREC Study, Canada, 2008 to 2011. Parameters were estimated using Dirichlet Bayesian weighted quantile sum regression models with 2 independent chains, each with 30,000 iterations, including 15,000 burn-in iterations. Models were adjusted for maternal age, parity, education, household income, household size, marital status, race/ethnicity, country of origin, folic acid supplementation, healthy eating index, study site, and fetal sex. 5MTHF, 5-methyltetrahydrofolate; UMFA, unmetabolized folic acid; NMF, nonmethylated folate; NO_2_, nitrogen dioxide; O_3_, ozone; PM_2.5_, particulate matter 2.5; SO_2_, sulfur dioxide.

The air pollution mixture was not associated with plasma total folate in the first trimester [–1.3 nmol/L, 95% confidence interval (CI): –7.3, 5.1 nmol/L] but was positively associated with plasma total folate in the third trimester (12.0 nmol/L, 95% CI: 1.5, 22.7 nmol/L) (Supplemental Table S4, Figure 1). In the latter model, O_3_ and PM_2.5_ were the dominant components (0.4 and 0.26, respectively) (Supplemental Table 4, Figure 1). In folate vitamer proportion models, the mixture was not associated with proportions in the first trimester but was negatively associated with %5MTHF (–2.8, 95% CI: –5.1, –0.7) and positively associated with %UMFA (13.4, 95% CI: 4.4, 23.0) in the third trimester (Supplemental Table 5, Figure 2). In the latter model, the dominant weight also was O_3_ (0.48) (Supplemental Table 5, Figure 2). In folate vitamer concentration models, the mixture was not associated with concentrations in the first trimester. In the third trimester, it was not associated with 5MTHF but was positively associated with UMFA (11.1, 95% CI: 2.2, 20.0) (Supplemental Table 6, Supplemental Figures 3–5). The dominant weight also was O_3_ (0.45) (Supplemental Table 6, Supplemental Figures 3–5).

**FIGURE 3:**
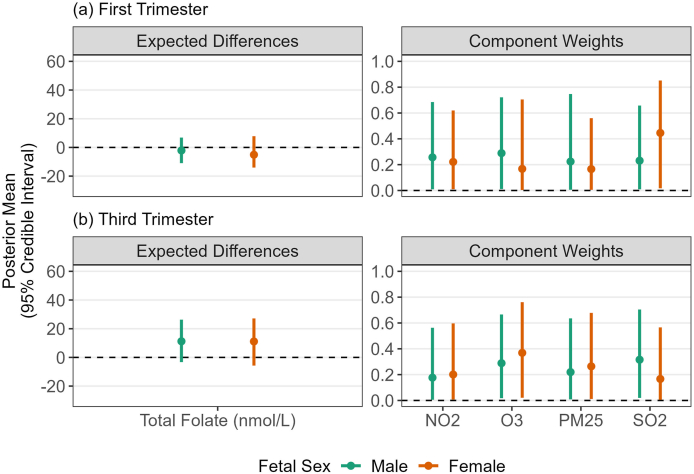
Mean (95% credible interval) posterior estimates of expected differences in total folate per 1-quartile difference in air pollution mixture and component weights by fetal sex in the first and third trimesters among pregnant women in Canada, MIREC Study, Canada, 2008 to 2011. Parameters were estimated using Bayesian weighted quantile sum regression models with 2 independent chains, each with 30,000 iterations, including 15,000 burn-in iterations. Models were adjusted for maternal age, parity, education, household income, household size, marital status, race/ethnicity, country of origin, folic acid supplementation, healthy eating index, and study site. NO_2_, nitrogen dioxide; O_3_, ozone; PM_2.5_, particulate matter 2.5; SO_2_, sulfur dioxide.

**FIGURE 4:**
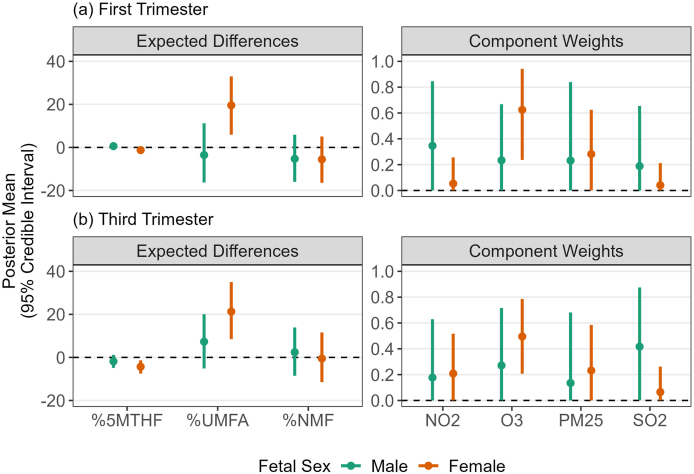
Mean (95% credible interval) posterior estimates of expected differences in folate vitamer proportions per 1-quartile difference in air pollution mixture and component weights by fetal sex in the first and third trimesters among pregnant women in Canada, MIREC Study, Canada, 2008 to 2011. Parameters were estimated using Dirichlet Bayesian weighted quantile sum regression models with 2 independent chains, each with 30,000 iterations, including 15,000 burn-in iterations. Models were adjusted for maternal age, parity, education, household income, household size, marital status, race/ethnicity, country of origin, folic acid supplementation, healthy eating index, and study site. 5MTHF, 5-methyltetrahydrofolate; UMFA, unmetabolized folic acid; NMF, nonmethylated folate; NO_2_, nitrogen dioxide; O_3_, ozone; PM_2.5_, particulate matter 2.5; SO_2_, sulfur dioxide.

**FIGURE 5:**
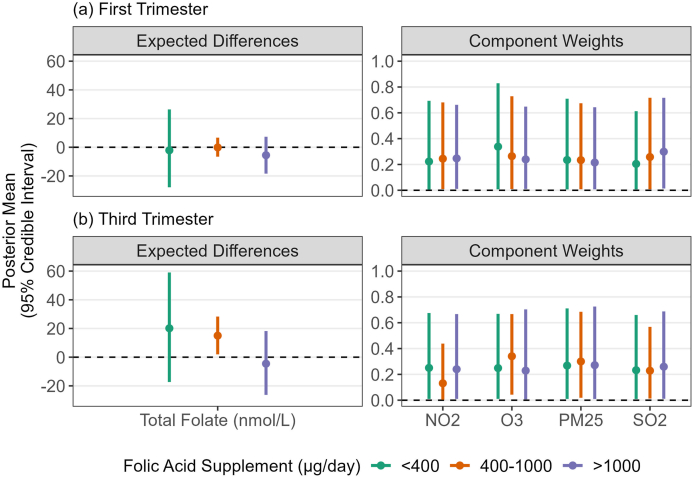
Mean (95% credible interval) posterior estimates of expected differences in total folate per 1-quartile difference in air pollution mixture and component weights by folic acid supplementation in the first and third trimesters among pregnant women in Canada, MIREC Study, Canada, 2008 to 2011. Parameters were estimated using Bayesian weighted quantile sum regression models with 2 independent chains, each with 30,000 iterations, including 15,000 burn-in iterations. Models were adjusted for maternal age, parity, education, household income, household size, marital status, race/ethnicity, country of origin, healthy eating index, study site, and fetal sex. NO_2_, nitrogen dioxide; O_3_, ozone; PM_2.5_, particulate matter 2.5; SO_2_, sulfur dioxide.

**FIGURE 6:**
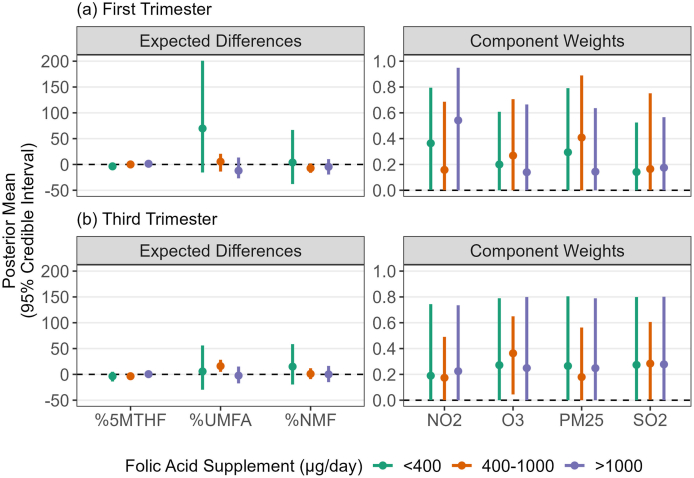
Mean (95% credible interval) posterior estimates of expected differences in folate vitamer proportions per 1-quartile difference in air pollution mixture and component weights by folic acid supplementation in the first and third trimesters among pregnant women in Canada, MIREC Study, Canada, 2008 to 2011. Parameters were estimated using Dirichlet Bayesian weighted quantile sum regression models with 2 independent chains, each with 30,000 iterations, including 15,000 burn-in iterations. Models were adjusted for maternal age, parity, education, household income, household size, marital status, race/ethnicity, country of origin, healthy eating index, study site, and fetal sex. 5MTHF, 5-methyltetrahydrofolate; UMFA, unmetabolized folic acid; NMF, nonmethylated folate; NO_2_, nitrogen dioxide; O_3_, ozone; PM_2.5_, particulate matter 2.5; SO_2_, sulfur dioxide.

In analyses stratified by fetal sex, only associations between the air pollution mixture and folate vitamer proportions appeared to vary by fetal sex (Supplemental Tables 4, 5, and 7; FIGURE 3, FIGURE 4, Supplemental Figures 6–8). Specifically, the negative association between the mixture and %5MTHF and the positive association between the mixture and %UMFA was stronger in women carrying female fetuses than in women carrying male fetuses in the first and third trimesters (Supplemental Table 5, FIGURE 3, FIGURE 4). In analyses stratified by FA supplementation, associations between the mixture and total folate in the third but not the first trimester attenuated with higher FA intake (Supplemental Tables 4 and 5, and Supplemental Table 8; Figure 5, Supplemental Figures 9–11). Specifically, in the third trimester, the expected difference in plasma total folate per-quartile difference in the mixture was 20.14 (–17.31, 59.05) for <400 μg/d, 15.01 (1.95, 28.30) for 400 to 1000 μg/d, and –4.53 (–26.28, 18.24) for >1000 μg/d (Supplemental Table 4). In the first trimester, associations between the mixture and %UMFA attenuated with higher FA intake (Supplemental Table 5, Figure 6).

## Discussion

In this prospective analysis of pregnant women, a mixture of NO_2_, O_3_, PM_2.5_, and SO_2_ air pollutants was positively associated with total folate concentrations and UMFA proportions and negatively associated with biologically active 5MTHF proportions. These associations with UMFA and 5MTHF proportions may be attributable to the increase in absolute UMFA concentrations as no association was observed with absolute 5MTHF concentrations. Associations were strongest in the third trimester and among women carrying female fetuses. O_3_ and PM_2.5_ appeared to drive these associations. At both visits, nearly all participants had plasma total folate above the cutoff associated with maximal NTD risk reduction. This suggests that air pollution mixtures including PM_2.5_ and O_3_ may influence folate status, including folate vitamer proportions, during pregnancy.

These findings add to a small but growing body of literature on environmental exposures and folate. Serum perfluoroalkyl and polyfluoroalkyl substances were negatively associated with RBC total folate among nonpregnant United States adolescents and adults in the NHANES [38]. Previous findings also suggest an inverse association between lead and folate among lead exposed workers and smoking and folate among Inuit women of childbearing age [20,39]. Whole blood lead was positively associated with serum homocysteine, which is inversely associated with circulating folate, among premenopausal women in Buffalo, New York [40]. These studies, however, were cross-sectional and could not address the potential for reverse causation given that FA supplementation has been shown to lower biomarkers of environmental exposures such as blood arsenic [41,42].

Folate is critical to developmental processes throughout pregnancy [43] and maternal hemodynamics and other factors affecting plasma folate change dramatically between the first and third trimesters [44]. Therefore, understanding how environmental exposures may interact with the folate system in early and late pregnancy is essential. Although no previous epidemiological study has investigated how gestational exposure to airborne pollutants impacts folate vitamer proportions, a recent computational study using a comparative toxicogenomic database identified 27 maternal environmental toxicants that may target folate receptors and transporters, with potential for disrupting folate metabolism even at low doses [45]. The sex-specific associations observed in our study, although exploratory, also warrants further investigation, especially given the observed sexually dimorphic effects of gestational exposure to air pollutants on child development [46,47]. Although these findings need to be replicated, existing evidence suggests that sex steroid hormones, particularly estrogen and progesterone, may influence folate metabolism. For instance, studies have shown that oral contraceptive hormones may disrupt folate metabolism in the cervical epithelium [48] and that testosterone may affect folate-dependent enzymes in the rat [49].

This study had multiple strengths. First, we conducted prospective analyses in a relatively large (*n* > 800) sample of pregnant women enrolled in 10 Canadian cities. Second, we assessed folate vitamers (5MTHF, UMFA, and NMF) as concentrations and proportions in addition to total folate to more completely assess how air pollution may influence maternal folate. Third, we measured plasma folate in both the first and third trimester, allowing us to examine associations in early and late pregnancy. Fourth, by adjusting for FA supplementation and a diet quality index in the first trimester, we held folate intake constant to better isolate in vivo effects of air pollution.

This study had some limitations as well. First, MIREC participants tended to be older and more affluent than Canadian women of reproductive age in general [23]. Additionally, our exposure assessment performed better in FSAs ≤20 km^2^ and we excluded many rural participants. Therefore, our results may not generalize to all pregnant women in Canada. Second, FSA-level estimates of air pollution concentrations may not be sufficient for assessing more spatially heterogenous pollutants such as NO_2_ and SO_2_. Additionally, we did not account for indoor air pollution sources. In our study, O_3_ and PM_2.5_ appeared to drive associations between the air pollution mixture and folate status, but future studies with alternative exposure assessment methods should consider NO_2_, SO_2_, and indoor air pollution sources as well. Third, maternal folate was measured in nonfasting plasma samples, which may result in higher intraindividual variability in folate vitamer concentrations. Additionally, plasma samples were stored at –80°C for 8 to 11 y before LC-MS/MS analysis. Some degradation in folate vitamers was expected [50], although rank-order among participants likely was unchanged. Finally, our diet quality index is an imperfect measure of dietary folate intake. FA supplementation and diet quality in the first trimester may not accurately measure FA or dietary folate intake in the third trimester. In preliminary analyses, however, diet quality in the first trimester was positively associated with plasma total folate in the first and third trimesters among women taking <400 μg/d of FA. Moreover, a recent study found that the healthy eating index, which is similar to our index, was consistent from early pregnancy to 1-y postpartum among women receiving obstetric care in Chapel Hill, North Carolina [51]. Nonetheless, control for folate intake may be limited, especially in the third trimester. However, we have no reason to believe folate intake was associated with air pollution, which would be necessary for it to confound our associations.

In conclusion, our analysis suggests that air pollution, especially PM_2.5_ and O_3_, may influence maternal folate in pregnancy. Associations were most pronounced in the third trimester and among women carrying female fetuses. Because prenatal maternal folate is important to neurodevelopment, and other studies have suggested that FA supplementation can modify associations between air pollution and autism, further research should examine these associations in other settings to see whether the relationships we observed are consistent and how they might vary by participant characteristics. At this stage, we believe new lines of research on FA supplementation and folate metabolism in pregnancy are needed to promote children’s neurodevelopment.

## Author contributions

The authors’ responsibilities were as follows – TJSS: performed the statistical analysis and drafted the paper; HS: developed software used for the statistical analysis; JMB, AJM, EL, MJ, AMS-R, RJ, MF, RTZ, JAM, TEA, BPL: designed or conducted the research; YO: designed and conducted the research, codrafted the paper, and obtained funding; and all authors: have read and approved the final manuscript and have primary responsibility for the final content of the paper.

## Data availability

Data described in the manuscript can be made available upon request pending application and approval by MIREC. See https://www.mirec-canada.ca/en/explore-the-mirec-research-platform. Analytic code will be made publicly and freely available on GitHub. See https://www.github.com/tylerjssmith/air_folate_mirec.

## Funding

The Maternal-Infant Research on Environmental Chemicals (MIREC) Study (http://www.mirec-canada.ca) was supported by the Chemicals Management Plan at Health Canada, the Ontario Ministry of the Environment, and the Canadian Institutes for Health Research (Open Operating grant 81285). This research was supported by the National Institute of Environmental Health Sciences (NIEHS) (R01ES032552). TJSS was supported by NIEHS (T32HD049311). Folate vitamer analysis was supported by Health Canada A-base (AJM) and NIEHS (JMB; R01ES024381).

## Conflict of interest

AMS-R is an Editorial Board Member for Current Developments in Nutrition and played no role in the Journal’s evaluation of the manuscript. The other authors report no conflicts of interest.
