# Supplementary material for: Air Pollution Mixture and Folate Status Indicators among Pregnant Women in Canada: Maternal-Infant Research on Environmental Chemicals (MIREC) Study, 2008 to 2011
Source: Curr Dev Nutr. 2025 Dec 10;10(7):107617. doi: 10.1016/j.cdnut.2025.107617 (PMC13355500; doi:10.1016/j.cdnut.2025.107617)
Supplement: Multimedia component 1 [file mmc1.docx]

Smith et al., Air Pollution Mixture and Folate Status Indicators among Pregnant Women in Canada: Maternal-Infant Research on Environmental Chemicals (MIREC) Study, 2008-2011

Supplemental Tables and Figures

[Table S1. Plasma Total Folate and Folate Vitamer Proportions in the First Trimester by Participant Characteristics, MIREC Study, Canada, 2008-2011 3](#_Toc213668011)

[Table S2. Plasma Total Folate and Folate Vitamer Proportions in the Third Trimester by Participant Characteristics, MIREC Study, Canada, 2008-2011 10](#_Toc213668012)

[Table S3. Median (Interquartile Range) Air Pollution Exposures in the First and Third Trimesters among Pregnant Women, MIREC Study, Canada, 2008-2011 16](#_Toc213668013)

[Table S4. Mean (95% Credible Interval) Posterior Estimates of Expected Differences in Plasma Total Folate per 1-Quartile Difference in Air Pollution Mixture and Component Weights in the First and Third Trimesters among Pregnant Women in Canada, MIREC Study, Canada, 2008-2011 17](#_Toc213668014)

[Table S5. Mean (95% Credible Interval) Posterior Estimates of Expected Differences in Folate Vitamer Proportions per 1-Quartile Difference in Air Pollution Mixture and Component Weights in the First and Third Trimesters among Pregnant Women in Canada, MIREC Study, Canada, 2008-2011 19](#_Toc213668015)

[Table S6. Mean (95% Credible Interval) Posterior Estimates of Expected Differences (nmol/L) in Folate Vitamer Concentrations per 1-Quartile Difference in Air Pollution Mixture and Component Weights in the First and Third Trimesters among Pregnant Women in Canada, MIREC Study, Canada, 2008-2011 21](#_Toc213668016)

[Table S7. Mean (95% Credible Interval) Posterior Estimates of Expected Differences (nmol/L) in Plasma Total Folate and Folate Vitamers per 1-Quartile Difference in Air Pollution Mixture and Component Weights in the First and Third Trimesters among Pregnant Women in Canada by Fetal Sex, MIREC Study, Canada, 2008-2011 22](#_Toc213668017)

[Table S8. Mean (95% Credible Interval) Posterior Estimates of Expected Differences (nmol/L) in Plasma Total Folate and Folate Vitamers per 1-Quartile Difference in Air Pollution Mixture and Component Weights in the First and Third Trimesters among Pregnant Women in Canada by Folic Acid Supplementation, MIREC Study, Canada, 2008-2011 24](#_Toc213668018)

[Figure S1. Plasma Total Folate by Diet Quality Index, Stratified by Folic Acid Supplementation, in the First and Third Trimesters, MIREC, Canada, 2008-2011 26](#_Toc213668019)

[Figure S2. Inclusion of Participants in Analysis of Air Pollution Mixture and Plasma Folate among Pregnant Women, MIREC, Canada, 2008-2011 27](#_Toc213668020)

[Figure S3. Mean (95% Credible Interval) Posterior Estimates of Expected Differences and Component Weights for Air Pollution and Plasma 5MTHF Concentrations in the First and Third Trimesters among Pregnant Women in Canada, MIREC Study, Canada, 2008-2011 28](#_Toc213668021)

[Figure S4. Mean (95% Credible Interval) Posterior Estimates of Expected Differences and Component Weights for Air Pollution and Plasma UMFA Concentrations in the First and Third Trimesters among Pregnant Women in Canada, MIREC Study, Canada, 2008-2011 29](#_Toc213668022)

[Figure S5. Mean (95% Credible Interval) Posterior Estimates of Expected Differences and Component Weights for Air Pollution and Plasma NMF Concentrations in the First and Third Trimesters among Pregnant Women in Canada, MIREC Study, Canada, 2008-2011 30](#_Toc213668023)

[Figure S6. Mean (95% Credible Interval) Posterior Estimates of Expected Differences and Component Weights for Air Pollution and Plasma 5MTHF Concentrations in the First and Third Trimesters among Pregnant Women by Fetal Sex in Canada, MIREC Study, Canada, 2008-2011 31](#_Toc213668024)

[Figure S7. Mean (95% Credible Interval) Posterior Estimates of Expected Differences and Component Weights for Air Pollution and Plasma UMFA Concentrations in the First and Third Trimesters among Pregnant Women by Fetal Sex in Canada, MIREC Study, Canada, 2008-2011 32](#_Toc213668025)

[Figure S8. Mean (95% Credible Interval) Posterior Estimates of Expected Differences and Component Weights for Air Pollution and Plasma NMF Concentrations in the First and Third Trimesters among Pregnant Women by Fetal Sex in Canada, MIREC Study, Canada, 2008-2011 33](#_Toc213668026)

[Figure S9. Mean (95% Credible Interval) Posterior Estimates of Mixture Associations and Component Weights for Air Pollution and Plasma 5MTHF Concentrations in the First and Third Trimesters among Pregnant Women by Folic Acid Supplementation in Canada, MIREC Study, Canada, 2008-2011 34](#_Toc213668027)

[Figure S10. Mean (95% Credible Interval) Posterior Estimates of Mixture Associations and Component Weights for Air Pollution and Plasma UMFA Concentrations in the First and Third Trimesters among Pregnant Women by Folic Acid Supplementation in Canada, MIREC Study, Canada, 2008-2011 35](#_Toc213668028)

[Figure S11. Mean (95% Credible Interval) Posterior Estimates of Mixture Associations and Component Weights for Air Pollution and Plasma NMF Concentrations in the First and Third Trimesters among Pregnant Women by Folic Acid Supplementation in Canada, MIREC Study, Canada, 2008-2011 36](#_Toc213668029)

# Table S1. Plasma Total Folate and Folate Vitamer Proportions in the First Trimester by Participant Characteristics, MIREC Study, Canada, 2008-2011

|  | Singleton Pregnancies with Complete Plasma Folate Data | | | | |
| --- | --- | --- | --- | --- | --- |
|  | n (%) | Total Folate (nmol/L) | 5MTHF (%) | UMFA (%) | NMF (%) |
| Overall | 1802 (100) | 95.7 (78.0, 117.0) | 93.8 (86.0, 96.6) | 2.8 (1.0, 10.0) | 2.2 (1.4, 3.3) |
| Age |  |  |  |  |  |
| ≤30 | 687 (38) | 91.1 ( 73.5, 112.4) | 94.0 (87.0, 96.5) | 2.6 (1.1, 9.0) | 2.2 (1.4, 3.4) |
| 31-35 | 616 (34) | 99.3 ( 79.7, 119.7) | 93.9 (85.3, 96.7) | 2.7 ( 1.0, 10.5) | 2.2 (1.4, 3.2) |
| ≥36 | 499 (28) | 98.9 ( 81.3, 121.2) | 93.6 (85.1, 96.8) | 3.1 ( 1.1, 10.6) | 2.1 (1.5, 3.3) |
| Parity |  |  |  |  |  |
| 0 | 794 (44) | 98.6 ( 80.5, 122.1) | 93.4 (85.7, 96.4) | 3.4 ( 1.2, 10.8) | 2.1 (1.4, 3.2) |
| 1 | 727 (40) | 94.8 ( 76.9, 113.9) | 94.1 (86.4, 96.7) | 2.5 (0.9, 8.8) | 2.2 (1.5, 3.5) |
| ≥2 | 281 (16) | 91.0 ( 72.4, 111.1) | 94.4 (87.2, 96.9) | 2.0 (0.9, 9.5) | 2.2 (1.5, 3.4) |
| Education |  |  |  |  |  |
| High school diploma or less | 523 (29) | 93.6 ( 73.7, 117.1) | 94.1 (86.2, 96.6) | 2.4 ( 0.9, 10.2) | 2.2 (1.4, 3.4) |
| College or trade school | 459 (25) | 98.8 ( 81.2, 118.7) | 93.7 (84.6, 96.7) | 3.0 ( 1.1, 10.7) | 2.1 (1.4, 3.2) |
| University degree | 159 (9) | 87.7 ( 66.3, 111.4) | 92.7 (83.4, 96.4) | 2.9 (1.2, 9.6) | 2.2 (1.5, 3.6) |
| Graduate degree | 659 (37) | 97.6 ( 80.2, 118.7) | 93.9 (87.2, 96.5) | 2.8 (1.0, 9.7) | 2.2 (1.4, 3.2) |
| Missing | 2 (0) | 76.8 (75.6, 78.0) | 97.3 (97.1, 97.6) | 1.3 (0.9, 1.7) | 1.4 (1.2, 1.5) |
| Household income |  |  |  |  |  |
| 0-50 | 314 (17) | 89.2 ( 67.4, 111.9) | 93.8 (87.6, 96.4) | 2.6 (1.0, 8.7) | 2.2 (1.5, 3.6) |
| 50-80 | 362 (20) | 94.9 ( 76.7, 118.0) | 93.8 (87.5, 96.6) | 2.8 (1.0, 9.3) | 2.1 (1.4, 3.3) |
| 80-100 | 357 (20) | 97.8 ( 80.2, 119.9) | 94.1 (84.7, 96.5) | 2.6 ( 1.1, 11.7) | 2.2 (1.5, 3.3) |
| >100 | 691 (38) | 97.1 ( 80.7, 117.7) | 93.9 (85.4, 96.7) | 2.8 (1.0, 9.8) | 2.1 (1.3, 3.2) |
| Missing | 78 (4) | 97.7 ( 75.5, 127.0) | 92.2 (82.2, 96.3) | 3.0 ( 1.3, 12.6) | 2.4 (1.4, 3.5) |

| Household size |  |  |  |  |  |
| --- | --- | --- | --- | --- | --- |
| 1 | 72 (4) | 105.7 ( 86.4, 136.7) | 90.7 (77.7, 96.0) | 4.3 ( 1.5, 20.9) | 2.2 (1.3, 3.1) |
| 2 | 746 (41) | 99.3 ( 80.5, 121.9) | 93.4 (85.8, 96.4) | 3.5 ( 1.2, 10.6) | 2.1 (1.4, 3.2) |
| 3 | 687 (38) | 94.3 ( 76.9, 112.7) | 94.4 (87.4, 96.8) | 2.4 (0.9, 8.2) | 2.2 (1.5, 3.5) |
| ≥4 | 277 (15) | 90.2 ( 74.3, 109.8) | 94.4 (86.3, 96.8) | 2.1 (1.0, 9.5) | 2.2 (1.4, 3.6) |
| Missing | 20 (1) | 90.9 ( 76.9, 118.2) | 90.7 (85.1, 97.4) | 5.1 ( 1.0, 11.9) | 1.9 (1.3, 3.0) |
| Marital status |  |  |  |  |  |
| Married or partnered | 1712 (95) | 95.6 ( 78.2, 117.2) | 93.9 (86.1, 96.6) | 2.7 (1.0, 9.8) | 2.2 (1.4, 3.3) |
| Not married | 90 (5) | 97.1 ( 59.0, 121.4) | 91.9 (84.8, 96.4) | 3.2 ( 1.2, 11.9) | 2.3 (1.1, 3.3) |
| Race/ethnicity |  |  |  |  |  |
| White | 1500 (83) | 96.0 ( 78.3, 117.5) | 93.8 (85.7, 96.5) | 2.8 ( 1.1, 10.6) | 2.1 (1.4, 3.2) |
| Non-white | 302 (17) | 92.4 ( 75.7, 117.8) | 94.0 (87.4, 96.9) | 2.3 (0.9, 8.9) | 2.4 (1.4, 3.7) |
| Country of origin |  |  |  |  |  |
| Canada or USA | 1538 (85) | 95.3 ( 77.8, 117.0) | 93.8 (86.0, 96.6) | 2.8 (1.1, 9.8) | 2.1 (1.4, 3.3) |
| Elsewhere | 264 (15) | 97.4 ( 78.8, 120.3) | 94.4 (86.3, 96.8) | 2.4 ( 0.9, 10.8) | 2.2 (1.3, 3.2) |
| Folic acid supplementation |  |  |  |  |  |
| <400 | 127 (7) | 73.7 (54.9, 90.1) | 94.9 (88.9, 96.9) | 2.1 (0.8, 5.0) | 2.5 (1.5, 3.8) |
| 400-1000 | 1231 (68) | 95.4 ( 78.2, 115.1) | 93.8 (85.8, 96.5) | 2.9 ( 1.1, 10.3) | 2.2 (1.4, 3.3) |
| >1000 | 444 (25) | 102.6 ( 82.7, 128.0) | 93.6 (85.5, 96.8) | 2.8 ( 1.0, 11.6) | 2.1 (1.3, 3.2) |
| Healthy eating index |  |  |  |  |  |
| Tertile 1 | 567 (31) | 94.8 ( 75.1, 120.7) | 93.9 (84.1, 96.7) | 2.7 ( 1.0, 12.0) | 2.1 (1.3, 3.3) |
| Tertile 2 | 564 (31) | 95.0 ( 78.4, 114.4) | 94.0 (87.7, 96.4) | 2.8 (1.2, 8.4) | 2.2 (1.4, 3.3) |
| Tertile 3 | 550 (31) | 97.2 ( 81.1, 117.2) | 93.5 (86.3, 96.6) | 2.7 ( 1.0, 10.3) | 2.1 (1.5, 3.2) |
| Missing | 121 (7) | 93.4 ( 78.3, 118.3) | 94.0 (89.2, 96.6) | 2.7 (1.0, 7.1) | 2.3 (1.7, 3.7) |

| Fetal sex |  |  |  |  |  |
| --- | --- | --- | --- | --- | --- |
| Male | 933 (52) | 95.7 ( 78.2, 116.8) | 93.7 (86.7, 96.6) | 2.9 (1.1, 9.4) | 2.2 (1.4, 3.4) |
| Female | 819 (45) | 96.2 ( 77.8, 118.5) | 93.9 (85.3, 96.6) | 2.7 ( 1.0, 11.4) | 2.1 (1.4, 3.2) |
| Missing | 50 (3) | 88.8 ( 74.9, 103.6) | 95.0 (89.4, 96.4) | 2.4 (1.3, 5.2) | 2.6 (1.9, 3.2) |

*Table S1 continued*

|  | Singleton Pregnancies with Complete Data | | | | |
| --- | --- | --- | --- | --- | --- |
|  | n (%) | Total Folate (nmol/L) | 5MTHF (%) | UMFA (%) | NMF (%) |
| Overall | 927 (100) | 96.9 (78.6, 119.0) | 93.9 (87.0, 96.8) | 2.6 (0.9, 9.5) | 2.1 (1.3, 3.3) |
| Age |  |  |  |  |  |
| ≤30 | 321 (35) | 89.3 ( 73.0, 110.7) | 94.0 (87.7, 96.5) | 2.5 (1.0, 8.3) | 2.1 (1.3, 3.2) |
| 31-35 | 327 (35) | 100.8 ( 82.6, 124.3) | 93.7 (84.0, 96.8) | 2.7 ( 0.9, 11.5) | 2.1 (1.2, 3.5) |
| ≥36 | 279 (30) | 99.5 ( 81.6, 120.1) | 94.0 (87.4, 96.9) | 2.6 (1.0, 9.0) | 2.2 (1.4, 3.3) |
| Parity |  |  |  |  |  |
| 0 | 411 (44) | 99.3 ( 80.5, 124.9) | 93.4 (85.7, 96.5) | 3.2 ( 1.1, 11.0) | 2.1 (1.2, 3.1) |
| 1 | 368 (40) | 95.5 ( 78.0, 114.4) | 94.4 (87.5, 96.9) | 2.3 (0.8, 7.7) | 2.2 (1.4, 3.6) |
| ≥2 | 148 (16) | 94.8 ( 75.0, 110.2) | 94.7 (87.6, 97.1) | 2.1 (0.9, 9.0) | 1.9 (1.3, 3.1) |
| Education |  |  |  |  |  |
| High school diploma or less | 219 (24) | 96.9 ( 74.4, 124.1) | 94.8 (86.3, 96.8) | 2.1 (0.9, 7.8) | 2.1 (1.3, 3.3) |
| College or trade school | 283 (31) | 97.1 ( 80.8, 115.5) | 93.7 (85.0, 96.8) | 2.9 ( 1.0, 10.7) | 2.1 (1.3, 3.3) |
| University degree | 69 (7) | 88.4 ( 65.9, 110.6) | 93.1 (88.0, 96.8) | 2.5 (1.1, 9.0) | 2.0 (1.3, 3.6) |
| Graduate degree | 356 (38) | 99.0 ( 80.8, 118.6) | 94.2 (87.6, 96.7) | 2.5 (0.9, 8.9) | 2.1 (1.2, 3.2) |
| Missing | 0 (0) | n/a | n/a | n/a | n/a |
| Household income |  |  |  |  |  |
| 0-50 | 165 (18) | 93.9 ( 67.2, 112.4) | 93.2 (86.3, 96.2) | 2.9 ( 1.0, 11.3) | 2.1 (1.3, 3.6) |
| 50-80 | 178 (19) | 95.3 ( 78.3, 117.6) | 94.3 (88.0, 96.6) | 2.7 (0.9, 8.2) | 2.1 (1.3, 3.2) |
| 80-100 | 186 (20) | 100.2 ( 80.7, 120.0) | 94.4 (85.3, 96.9) | 2.1 (1.0, 9.5) | 2.2 (1.3, 3.4) |
| >100 | 398 (43) | 97.2 ( 80.8, 120.8) | 94.0 (87.0, 96.9) | 2.6 (0.9, 9.5) | 2.0 (1.2, 3.1) |
| Missing | 0 (0) | n/a | n/a | n/a | n/a |

| Household size |  |  |  |  |  |
| --- | --- | --- | --- | --- | --- |
| 1 | 40 (4) | 115.0 ( 94.3, 138.0) | 89.3 (77.7, 96.0) | 7.4 ( 1.8, 20.9) | 1.9 (1.0, 2.9) |
| 2 | 385 (42) | 97.9 ( 79.4, 124.0) | 93.8 (87.1, 96.7) | 2.8 (1.0, 9.4) | 2.0 (1.2, 3.1) |
| 3 | 365 (39) | 94.7 ( 78.1, 113.9) | 94.1 (87.5, 96.8) | 2.3 (0.8, 7.8) | 2.3 (1.4, 3.6) |
| ≥4 | 137 (15) | 94.9 ( 78.0, 109.9) | 94.2 (87.1, 97.0) | 2.3 (1.0, 9.5) | 1.9 (1.3, 3.1) |
| Missing | 0 (0) | n/a | n/a | n/a | n/a |
| Marital status |  |  |  |  |  |
| Married or partnered | 877 (95) | 96.6 ( 78.7, 117.5) | 94.0 (87.1, 96.8) | 2.5 (0.9, 9.4) | 2.1 (1.3, 3.3) |
| Not married | 50 (5) | 99.2 ( 64.7, 127.5) | 91.0 (85.7, 96.4) | 3.2 ( 1.5, 11.4) | 1.9 (1.0, 3.5) |
| Race/ethnicity |  |  |  |  |  |
| White | 737 (80) | 97.3 ( 79.2, 119.7) | 93.7 (85.7, 96.6) | 2.8 ( 1.0, 10.8) | 2.1 (1.3, 3.2) |
| Non-white | 190 (20) | 94.5 ( 77.1, 113.1) | 94.8 (89.9, 97.4) | 1.7 (0.8, 5.7) | 2.2 (1.3, 3.7) |
| Country of origin |  |  |  |  |  |
| Canada or USA | 740 (80) | 96.5 ( 78.2, 118.7) | 93.9 (86.3, 96.7) | 2.6 (1.0, 9.5) | 2.1 (1.3, 3.3) |
| Elsewhere | 187 (20) | 97.7 ( 83.4, 119.3) | 94.4 (87.7, 97.0) | 2.4 (0.8, 9.4) | 2.2 (1.3, 3.2) |
| Folic acid supplementation |  |  |  |  |  |
| <400 | 59 (6) | 73.7 (57.0, 95.5) | 95.4 (91.4, 97.1) | 1.7 (0.7, 4.0) | 2.5 (1.6, 4.6) |
| 400-1000 | 623 (67) | 96.5 ( 79.9, 114.7) | 93.8 (86.1, 96.7) | 2.6 ( 0.9, 10.3) | 2.1 (1.2, 3.2) |
| >1000 | 245 (26) | 103.1 ( 81.4, 127.5) | 94.1 (86.1, 96.8) | 2.6 (1.0, 9.7) | 2.1 (1.3, 3.2) |
| Healthy eating index |  |  |  |  |  |
| Tertile 1 | 276 (30) | 95.4 ( 75.7, 121.8) | 94.1 (85.2, 97.0) | 2.4 ( 0.8, 10.9) | 2.0 (1.1, 3.3) |
| Tertile 2 | 306 (33) | 97.1 ( 79.4, 114.9) | 94.0 (88.0, 96.3) | 2.8 (1.1, 9.1) | 2.2 (1.3, 3.3) |
| Tertile 3 | 345 (37) | 97.3 ( 81.6, 117.8) | 93.8 (87.2, 96.9) | 2.6 (0.9, 9.3) | 2.1 (1.4, 3.3) |
| Missing | 0 (0) | n/a | n/a | n/a | n/a |

| Fetal sex |  |  |  |  |  |
| --- | --- | --- | --- | --- | --- |
| Male | 489 (53) | 97.7 ( 79.8, 119.7) | 94.0 (87.6, 96.9) | 2.6 (0.9, 8.6) | 2.1 (1.3, 3.4) |
| Female | 438 (47) | 95.6 ( 77.9, 117.2) | 93.9 (85.6, 96.6) | 2.6 ( 0.9, 11.0) | 2.1 (1.2, 3.2) |
| Missing | 0 (0) | n/a | n/a | n/a | n/a |

# Abbreviations: 5MTHF, 5-methyltetrahydrofolate; UMFA, unmetabolized folic acid; NMF, non-methylated folate

# Table S2. Plasma Total Folate and Folate Vitamer Proportions in the Third Trimester by Participant Characteristics, MIREC Study, Canada, 2008-2011

|  | Singleton Pregnancies with Complete Plasma Folate Data | | | | |
| --- | --- | --- | --- | --- | --- |
|  | n (%) | Total Folate (nmol/L) | 5MTHF (%) | UMFA (%) | NMF (%) |
| Overall | 1568 (100) | 98.2 (74.2, 133.0) | 81.9 (70.1, 87.3) | 9.1 (5.2, 20.1) | 6.3 (4.3, 9.2) |
| Age |  |  |  |  |  |
| ≤30 | 598 (38) | 94.5 ( 67.7, 123.5) | 82.5 (72.5, 87.7) | 8.8 ( 5.1, 17.1) | 6.4 (4.3, 9.6) |
| 31-35 | 536 (34) | 101.5 ( 75.9, 138.3) | 81.7 (66.5, 87.0) | 9.1 ( 5.3, 24.6) | 6.2 (4.3, 9.0) |
| ≥36 | 434 (28) | 103.0 ( 77.3, 151.1) | 81.6 (68.7, 87.1) | 9.3 ( 5.1, 22.1) | 6.4 (4.5, 9.0) |
| Parity |  |  |  |  |  |
| 0 | 697 (44) | 107.3 ( 85.3, 148.6) | 82.1 (67.1, 87.3) | 9.4 ( 5.4, 24.9) | 5.9 (4.2, 8.5) |
| 1 | 629 (40) | 92.7 ( 67.7, 123.6) | 82.1 (72.5, 87.4) | 8.6 ( 4.8, 16.3) | 6.7 (4.5, 9.5) |
| ≥2 | 242 (15) | 84.7 ( 56.5, 122.5) | 81.2 (69.7, 86.1) | 9.1 ( 5.3, 19.1) | 6.6 ( 4.6, 10.7) |
| Education |  |  |  |  |  |
| High school diploma or less | 447 (29) | 92.0 ( 65.8, 124.3) | 82.5 (72.2, 87.1) | 8.5 ( 5.2, 17.7) | 6.6 (4.7, 9.7) |
| College or trade school | 408 (26) | 103.6 ( 81.4, 139.8) | 81.1 (67.4, 87.1) | 10.0 ( 5.4, 23.1) | 6.1 (4.1, 8.8) |
| University degree | 127 (8) | 81.4 ( 47.9, 116.0) | 81.0 (72.4, 87.0) | 9.3 ( 5.0, 16.1) | 7.4 ( 4.5, 11.9) |
| Graduate degree | 584 (37) | 101.7 ( 77.3, 142.5) | 82.2 (68.4, 87.8) | 8.9 ( 5.0, 23.3) | 6.2 (4.2, 8.8) |
| Missing | 2 (0) | 58.8 (48.4, 69.1) | 77.8 (76.4, 79.2) | 13.5 (12.8, 14.1) | 8.7 (8.0, 9.5) |
| Household income |  |  |  |  |  |
| 0-50 | 260 (17) | 87.1 ( 53.0, 126.5) | 81.6 (70.0, 87.4) | 8.8 ( 5.0, 18.1) | 6.9 ( 4.7, 11.3) |
| 50-80 | 329 (21) | 98.3 ( 74.7, 133.4) | 81.0 (68.5, 86.6) | 9.9 ( 5.9, 19.9) | 6.5 (4.5, 9.7) |
| 80-100 | 311 (20) | 103.8 ( 77.4, 149.0) | 82.4 (67.7, 87.1) | 9.1 ( 4.7, 24.8) | 6.1 (4.3, 8.7) |
| >100 | 606 (39) | 100.6 ( 78.2, 131.6) | 82.5 (71.6, 87.7) | 8.7 ( 5.1, 19.2) | 6.1 (4.2, 8.6) |
| Missing | 62 (4) | 86.3 ( 68.9, 134.7) | 80.8 (70.1, 86.7) | 10.1 ( 6.0, 21.6) | 6.5 (4.4, 8.2) |

| Household size |  |  |  |  |  |
| --- | --- | --- | --- | --- | --- |
| 1 | 62 (4) | 111.3 ( 77.0, 154.5) | 79.3 (67.4, 85.9) | 10.1 ( 4.1, 27.4) | 6.3 (3.8, 9.8) |
| 2 | 655 (42) | 106.3 ( 84.2, 144.9) | 82.2 (67.1, 87.3) | 9.5 ( 5.5, 24.6) | 6.0 (4.2, 8.3) |
| 3 | 593 (38) | 92.9 ( 67.9, 124.6) | 82.2 (71.5, 87.4) | 8.6 ( 4.8, 16.3) | 6.8 (4.5, 9.5) |
| ≥4 | 240 (15) | 87.7 ( 60.3, 119.0) | 81.3 (71.4, 87.0) | 8.9 ( 5.0, 19.7) | 6.5 ( 4.7, 10.4) |
| Missing | 18 (1) | 86.2 ( 69.7, 119.1) | 82.0 (75.3, 85.6) | 9.4 ( 6.2, 17.8) | 7.2 (6.0, 8.1) |
| Marital status |  |  |  |  |  |
| Married or partnered | 1495 (95) | 98.7 ( 74.9, 133.3) | 81.9 (70.1, 87.3) | 9.0 ( 5.2, 20.1) | 6.3 (4.3, 9.1) |
| Not married | 73 (5) | 78.4 ( 47.0, 123.4) | 81.3 (70.5, 87.5) | 9.4 ( 5.2, 19.1) | 7.1 ( 4.7, 10.6) |
| Race/ethnicity |  |  |  |  |  |
| White | 1306 (83) | 98.6 ( 74.9, 136.4) | 81.7 (69.4, 87.2) | 9.1 ( 5.1, 21.4) | 6.3 (4.3, 9.2) |
| Non-white | 262 (17) | 94.9 ( 66.8, 124.2) | 82.9 (73.7, 87.6) | 8.8 ( 5.7, 16.4) | 6.3 (4.6, 8.8) |
| Country of origin |  |  |  |  |  |
| Canada or USA | 1335 (85) | 98.0 ( 73.2, 131.4) | 81.8 (69.6, 87.2) | 9.1 ( 5.2, 20.2) | 6.5 (4.4, 9.4) |
| Elsewhere | 233 (15) | 102.0 ( 76.3, 140.2) | 82.7 (72.0, 88.1) | 8.9 ( 5.4, 18.5) | 5.9 (4.3, 8.2) |
| Folic acid supplementation |  |  |  |  |  |
| <400 | 113 (7) | 61.7 (40.8, 90.8) | 79.7 (74.0, 84.9) | 9.1 ( 5.2, 14.2) | 9.0 ( 6.1, 13.1) |
| 400-1000 | 1081 (69) | 99.5 ( 75.6, 134.6) | 82.3 (68.9, 87.5) | 9.0 ( 5.2, 22.2) | 6.1 (4.2, 8.9) |
| >1000 | 374 (24) | 103.6 ( 79.5, 144.1) | 81.7 (70.3, 87.3) | 9.3 ( 5.0, 19.8) | 6.5 (4.8, 8.9) |
| Healthy eating index |  |  |  |  |  |
| Tertile 1 | 515 (33) | 93.6 ( 62.5, 125.1) | 82.1 (70.3, 87.3) | 8.8 ( 4.7, 18.2) | 6.7 ( 4.6, 10.4) |
| Tertile 2 | 526 (34) | 98.0 ( 76.3, 138.7) | 81.4 (69.8, 87.1) | 9.4 ( 5.5, 22.2) | 6.2 (4.3, 8.8) |
| Tertile 3 | 514 (33) | 103.4 ( 81.2, 137.9) | 82.4 (69.8, 87.5) | 9.2 ( 5.2, 20.9) | 6.1 (4.3, 8.6) |
| Missing | 13 (1) | 69.0 ( 48.8, 102.3) | 83.9 (77.3, 90.3) | 7.0 (5.9, 8.5) | 7.9 ( 5.6, 10.0) |

| Fetal sex |  |  |  |  |  |
| --- | --- | --- | --- | --- | --- |
| Male | 829 (53) | 98.0 ( 72.4, 132.6) | 81.8 (70.3, 87.4) | 9.1 ( 5.1, 19.7) | 6.3 (4.4, 9.1) |
| Female | 735 (47) | 98.8 ( 75.4, 134.6) | 82.0 (69.4, 87.3) | 9.0 ( 5.2, 22.2) | 6.3 (4.3, 9.3) |
| Missing | 4 (0) | 137.5 (113.2, 151.0) | 80.8 (76.5, 84.7) | 9.5 ( 5.5, 14.0) | 7.0 ( 5.5, 11.0) |

*Table S2 continued*

|  | Singleton Pregnancies with Complete Data | | | | |
| --- | --- | --- | --- | --- | --- |
|  | n (%) | Total Folate (nmol/L) | 5MTHF (%) | UMFA (%) | NMF (%) |
| Overall | 838 (100) | 99.6 (76.0, 135.0) | 82.1 (69.2, 87.5) | 8.9 (5.1, 20.1) | 6.2 (4.4, 9.0) |
| Age |  |  |  |  |  |
| ≤30 | 292 (35) | 90.8 ( 67.6, 119.9) | 81.8 (71.5, 87.6) | 9.5 ( 5.2, 17.5) | 6.3 (4.3, 9.4) |
| 31-35 | 303 (36) | 104.9 ( 81.0, 144.7) | 82.2 (65.5, 87.3) | 8.8 ( 5.2, 26.3) | 6.1 (4.3, 8.8) |
| ≥36 | 243 (29) | 105.8 ( 79.2, 147.6) | 82.3 (70.3, 87.7) | 8.3 ( 4.3, 21.2) | 6.4 (4.7, 9.1) |
| Parity |  |  |  |  |  |
| 0 | 379 (45) | 107.1 ( 84.8, 151.4) | 82.1 (65.5, 87.6) | 9.3 ( 5.1, 27.0) | 5.8 (4.2, 8.4) |
| 1 | 337 (40) | 92.9 ( 71.9, 122.2) | 82.5 (72.9, 87.9) | 8.2 ( 4.7, 15.3) | 6.6 (4.8, 9.4) |
| ≥2 | 122 (15) | 88.2 ( 56.8, 130.0) | 80.4 (67.6, 86.0) | 9.6 ( 5.9, 22.3) | 6.2 ( 4.5, 10.4) |
| Education |  |  |  |  |  |
| High school diploma or less | 205 (24) | 92.0 ( 65.3, 126.4) | 83.0 (70.5, 87.4) | 8.8 ( 5.1, 18.5) | 6.6 (4.9, 9.2) |
| College or trade school | 267 (32) | 104.9 ( 81.4, 142.0) | 81.5 (66.3, 87.2) | 9.5 ( 5.2, 25.2) | 6.0 (4.2, 8.6) |
| University degree | 53 (6) | 78.9 ( 47.6, 102.3) | 79.3 (71.6, 86.9) | 9.6 ( 6.0, 15.7) | 7.7 ( 4.4, 12.8) |
| Graduate degree | 313 (37) | 101.1 ( 77.5, 139.4) | 82.9 (69.7, 88.1) | 8.3 ( 4.7, 21.2) | 6.1 (4.3, 8.8) |
| Missing | 0 (0) | n/a | n/a | n/a | n/a |
| Household income |  |  |  |  |  |
| 0-50 | 151 (18) | 92.0 ( 53.9, 130.3) | 79.5 (67.2, 86.5) | 11.3 ( 5.6, 19.2) | 6.6 ( 4.5, 10.5) |
| 50-80 | 179 (21) | 93.5 ( 74.8, 125.5) | 81.0 (70.6, 87.4) | 9.7 ( 5.7, 17.3) | 6.5 (4.8, 9.4) |
| 80-100 | 150 (18) | 105.5 ( 77.4, 166.4) | 82.9 (66.4, 87.1) | 9.0 ( 4.6, 28.4) | 6.1 (3.8, 8.5) |
| >100 | 358 (43) | 102.5 ( 80.4, 137.3) | 83.6 (70.6, 88.1) | 8.2 ( 4.7, 20.4) | 6.1 (4.4, 8.5) |
| Missing | 0 (0) | n/a | n/a | n/a | n/a |

| Household size |  |  |  |  |  |
| --- | --- | --- | --- | --- | --- |
| 1 | 40 (5) | 111.6 ( 79.6, 149.9) | 80.2 (67.5, 86.8) | 9.3 ( 3.2, 27.3) | 5.9 (4.0, 9.2) |
| 2 | 354 (42) | 106.0 ( 83.9, 151.1) | 82.2 (65.8, 87.7) | 9.4 ( 5.2, 26.3) | 5.9 (4.1, 8.3) |
| 3 | 330 (39) | 93.2 ( 72.0, 123.6) | 82.6 (72.7, 87.5) | 8.2 ( 4.7, 15.4) | 6.6 (4.8, 9.4) |
| ≥4 | 114 (14) | 90.1 ( 58.2, 118.6) | 80.4 (67.6, 87.1) | 9.2 ( 5.5, 22.3) | 6.2 (4.8, 9.8) |
| Missing | 0 (0) | n/a | n/a | n/a | n/a |
| Marital status |  |  |  |  |  |
| Married or partnered | 792 (95) | 99.6 ( 76.4, 134.8) | 82.2 (69.5, 87.5) | 8.8 ( 5.0, 20.7) | 6.2 (4.4, 9.0) |
| Not married | 46 (5) | 99.0 ( 59.3, 133.3) | 81.5 (67.9, 87.6) | 9.4 ( 5.1, 20.9) | 6.8 ( 4.7, 10.1) |
| Race/ethnicity |  |  |  |  |  |
| White | 672 (80) | 99.9 ( 76.4, 137.6) | 81.7 (68.3, 87.5) | 9.0 ( 4.9, 23.6) | 6.2 (4.4, 9.1) |
| Non-white | 166 (20) | 98.1 ( 72.6, 123.9) | 83.1 (75.5, 87.6) | 8.7 ( 5.7, 15.7) | 6.3 (4.8, 8.5) |
| Country of origin |  |  |  |  |  |
| Canada or USA | 674 (80) | 98.2 ( 74.9, 131.7) | 82.1 (69.2, 87.4) | 8.9 ( 4.9, 20.6) | 6.4 (4.4, 9.2) |
| Elsewhere | 164 (20) | 104.0 ( 81.2, 149.9) | 82.4 (70.5, 88.1) | 8.8 ( 5.2, 21.1) | 5.9 (4.3, 8.4) |
| Folic acid supplementation |  |  |  |  |  |
| <400 | 55 (7) | 66.0 ( 45.5, 101.8) | 80.0 (72.1, 85.4) | 8.4 ( 5.1, 14.9) | 9.0 ( 6.2, 14.0) |
| 400-1000 | 570 (68) | 100.1 ( 76.8, 138.6) | 82.3 (67.3, 87.5) | 9.0 ( 5.2, 24.2) | 6.0 (4.1, 8.8) |
| >1000 | 213 (25) | 103.6 ( 82.2, 134.6) | 82.2 (71.9, 88.3) | 8.3 ( 4.5, 18.5) | 6.5 (4.9, 8.6) |
| Healthy eating index |  |  |  |  |  |
| Tertile 1 | 239 (29) | 94.5 ( 61.3, 130.5) | 82.1 (66.4, 87.6) | 8.6 ( 4.2, 21.6) | 6.6 ( 4.6, 10.0) |
| Tertile 2 | 283 (34) | 98.3 ( 77.1, 141.6) | 80.8 (70.0, 87.1) | 9.2 ( 5.3, 20.2) | 6.2 (4.3, 8.9) |
| Tertile 3 | 316 (38) | 103.7 ( 81.7, 135.2) | 83.3 (70.6, 87.8) | 8.7 ( 5.1, 19.4) | 6.0 (4.4, 8.5) |
| Missing | 0 (0) | n/a | n/a | n/a | n/a |

| Fetal sex |  |  |  |  |  |
| --- | --- | --- | --- | --- | --- |
| Male | 453 (54) | 100.5 ( 76.5, 136.5) | 82.3 (69.2, 87.5) | 8.9 ( 5.1, 19.7) | 6.3 (4.5, 8.8) |
| Female | 385 (46) | 99.3 ( 75.5, 130.3) | 82.0 (69.2, 87.5) | 8.9 ( 5.1, 23.2) | 6.1 (4.4, 9.1) |
| Missing | 0 (0) | n/a | n/a | n/a | n/a |

# Abbreviations: 5MTHF, 5-methyltetrahydrofolate; UMFA, unmetabolized folic acid; NMF, non-methylated folate

# Table S3. Median (Interquartile Range) Air Pollution Exposures in the First and Third Trimesters among Pregnant Women, MIREC Study, Canada, 2008-2011

| Air Pollutant | First Trimester | Third Trimester |
| --- | --- | --- |
| NO2 | 22.5 (13.3, 30.2) | 21.3 (13.6, 28.7) |
| O3 | 21.3 (18.2, 25.6) | 22.8 (19.4, 27.0) |
| PM25 | 10.2 ( 7.3, 13.4) | 9.9 ( 7.4, 13.9) |
| SO2 | 1.5 ( 0.9, 1.9) | 1.3 ( 0.9, 1.7) |

A participant's exposure was the mean of estimated daily concentrations at the participant's residences over 180 days preceding a study visit in the first trimester (6-<14 gestational weeks) or third trimester (32-34 weeks).

Abbreviations: NO2, nitrogen dioxide; O3, ozone; PM25, particulate matter 2.5; SO2, sulfur dioxide

# Table S4. Mean (95% Credible Interval) Posterior Estimates of Expected Differences in Plasma Total Folate per 1-Quartile Difference in Air Pollution Mixture and Component Weights in the First and Third Trimesters among Pregnant Women in Canada, MIREC Study, Canada, 2008-2011

| Stratum | Parameter | First Trimester | Third Trimester |
| --- | --- | --- | --- |
| Overall | Total Folate (nmol/L) | -1.31 (-7.25, 5.08) | 12.04 ( 1.54, 22.68) |
|  | NO2 | 0.26 ( 0.01, 0.71) | 0.15 ( 0.00, 0.46) |
|  | O3 | 0.23 ( 0.01, 0.67) | 0.40 ( 0.06, 0.73) |
|  | PM25 | 0.22 ( 0.01, 0.69) | 0.26 ( 0.01, 0.64) |
|  | SO2 | 0.29 ( 0.01, 0.74) | 0.20 ( 0.01, 0.52) |
| Males | Total Folate (nmol/L) | -2.09 (-10.87, 6.93) | 11.81 ( -2.44, 26.80) |
|  | NO2 | 0.26 ( 0.01, 0.68) | 0.17 ( 0.01, 0.55) |
|  | O3 | 0.29 ( 0.01, 0.72) | 0.28 ( 0.02, 0.66) |
|  | PM25 | 0.22 ( 0.01, 0.74) | 0.22 ( 0.01, 0.62) |
|  | SO2 | 0.23 ( 0.01, 0.65) | 0.32 ( 0.02, 0.71) |
| Females | Total Folate (nmol/L) | -4.45 (-13.63, 8.26) | 11.49 ( -4.90, 27.35) |
|  | NO2 | 0.22 ( 0.01, 0.62) | 0.20 ( 0.01, 0.60) |
|  | O3 | 0.18 ( 0.00, 0.72) | 0.37 ( 0.03, 0.75) |
|  | PM25 | 0.17 ( 0.00, 0.58) | 0.27 ( 0.01, 0.68) |
|  | SO2 | 0.42 ( 0.01, 0.84) | 0.16 ( 0.01, 0.55) |
| Folic Acid <400 µg/day | Total Folate (nmol/L) | -2.14 (-27.92, 26.37) | 20.14 (-17.31, 59.05) |
|  | NO2 | 0.22 ( 0.01, 0.69) | 0.25 ( 0.01, 0.68) |
|  | O3 | 0.34 ( 0.01, 0.83) | 0.25 ( 0.01, 0.67) |
|  | PM25 | 0.23 ( 0.01, 0.71) | 0.27 ( 0.01, 0.71) |
|  | SO2 | 0.20 ( 0.01, 0.61) | 0.23 ( 0.01, 0.66) |
| Folic Acid 400-1,000 µg/day | Total Folate (nmol/L) | -0.13 ( -6.58, 6.68) | 15.01 ( 1.95, 28.30) |
|  | NO2 | 0.24 ( 0.01, 0.68) | 0.13 ( 0.00, 0.44) |
|  | O3 | 0.26 ( 0.01, 0.73) | 0.34 ( 0.04, 0.67) |
|  | PM25 | 0.23 ( 0.01, 0.67) | 0.30 ( 0.02, 0.68) |
|  | SO2 | 0.26 ( 0.01, 0.72) | 0.23 ( 0.01, 0.57) |
| Folic Acid >1,000 µg/day | Total Folate (nmol/L) | -5.54 (-18.40, 7.30) | -4.53 (-26.28, 18.24) |
|  | NO2 | 0.25 ( 0.01, 0.66) | 0.24 ( 0.01, 0.67) |
|  | O3 | 0.24 ( 0.01, 0.65) | 0.23 ( 0.01, 0.70) |
|  | PM25 | 0.21 ( 0.01, 0.64) | 0.27 ( 0.01, 0.73) |
|  | SO2 | 0.30 ( 0.01, 0.72) | 0.26 ( 0.01, 0.69) |

Parameters were estimated using Bayesian weighted quantile sum (BWQS) regression models with two independent chains, each with 30,000 iterations, including 15,000 burn-in iterations. Models were adjusted for maternal age, parity, education, household income, household size, marital status, race/ethnicity, country of origin, folic acid supplementation, healthy eating index, study site, and fetal sex.

# Abbreviations: NO2, nitrogen dioxide; O3, ozone; PM25, particulate matter 2.5; SO2, sulfur dioxide

# Table S5. Mean (95% Credible Interval) Posterior Estimates of Expected Differences in Folate Vitamer Proportions per 1-Quartile Difference in Air Pollution Mixture and Component Weights in the First and Third Trimesters among Pregnant Women in Canada, MIREC Study, Canada, 2008-2011

| Stratum | Parameter | First Trimester | Third Trimester |
| --- | --- | --- | --- |
| Overall | %5MTHF | -0.30 ( -1.24, 0.99) | -2.78 ( -5.06, -0.69) |
|  | %UMFA | 9.83 ( -8.19, 20.48) | 13.35 ( 4.40, 23.03) |
|  | %NMF | 0.93 ( 0.85, 1.01) | 1.00 ( 0.92, 1.10) |
|  | NO2 | 0.08 ( 0.00, 0.62) | 0.21 ( 0.00, 0.52) |
|  | O3 | 0.36 ( 0.00, 0.74) | 0.48 ( 0.18, 0.79) |
|  | PM25 | 0.48 ( 0.00, 0.86) | 0.11 ( 0.00, 0.41) |
|  | SO2 | 0.07 ( 0.00, 0.44) | 0.20 ( 0.00, 0.49) |
| Males | %5MTHF | 0.60 ( -0.64, 1.79) | -1.84 ( -4.89, 1.18) |
|  | %UMFA | -3.52 (-16.32, 11.22) | 7.31 ( -5.13, 20.07) |
|  | %NMF | 0.95 ( 0.84, 1.06) | 1.02 ( 0.91, 1.14) |
|  | NO2 | 0.35 ( 0.00, 0.85) | 0.18 ( 0.00, 0.63) |
|  | O3 | 0.23 ( 0.00, 0.67) | 0.27 ( 0.00, 0.72) |
|  | PM25 | 0.23 ( 0.00, 0.84) | 0.14 ( 0.00, 0.68) |
|  | SO2 | 0.19 ( 0.00, 0.65) | 0.42 ( 0.00, 0.88) |
| Females | %5MTHF | -1.25 ( -2.52, 0.15) | -4.33 ( -7.51, -1.36) |
|  | %UMFA | 19.56 ( 5.94, 33.00) | 21.31 ( 8.50, 35.04) |
|  | %NMF | 0.94 ( 0.84, 1.05) | 0.99 ( 0.88, 1.12) |
|  | NO2 | 0.05 ( 0.00, 0.26) | 0.21 ( 0.00, 0.52) |
|  | O3 | 0.62 ( 0.24, 0.94) | 0.49 ( 0.21, 0.79) |
|  | PM25 | 0.28 ( 0.00, 0.62) | 0.23 ( 0.00, 0.58) |
|  | SO2 | 0.04 ( 0.00, 0.21) | 0.06 ( 0.00, 0.26) |
| Folic Acid <400 µg/day | %5MTHF | -3.66 (-10.05, 1.09) | -3.66 (-13.70, 5.00) |
|  | %UMFA | 69.72 (-15.51, 200.89) | 5.74 (-29.66, 55.95) |
|  | %NMF | 3.78 (-37.90, 66.80) | 15.03 (-19.54, 58.60) |
|  | NO2 | 0.36 ( 0.00, 0.79) | 0.19 ( 0.00, 0.74) |
|  | O3 | 0.20 ( 0.00, 0.61) | 0.27 ( 0.00, 0.79) |
|  | PM25 | 0.29 ( 0.00, 0.79) | 0.27 ( 0.00, 0.80) |
|  | SO2 | 0.14 ( 0.00, 0.52) | 0.27 ( 0.00, 0.80) |
| Folic Acid 400-1,000 µg/day | %5MTHF | 0.03 ( -1.30, 1.55) | -3.56 ( -6.45, -0.88) |
|  | %UMFA | 5.40 (-13.72, 20.65) | 16.03 ( 4.95, 28.26) |
|  | %NMF | -7.20 (-16.20, 2.11) | 0.99 ( -9.12, 11.87) |
|  | NO2 | 0.16 ( 0.00, 0.69) | 0.17 ( 0.00, 0.49) |
|  | O3 | 0.27 ( 0.00, 0.71) | 0.36 ( 0.04, 0.65) |
|  | PM25 | 0.41 ( 0.00, 0.89) | 0.18 ( 0.00, 0.56) |
|  | SO2 | 0.16 ( 0.00, 0.75) | 0.28 ( 0.00, 0.61) |
| Folic Acid >1,000 µg/day | %5MTHF | 1.33 ( -0.86, 2.95) | 0.45 ( -3.12, 3.88) |
|  | %UMFA | -12.02 (-26.87, 13.46) | -2.32 (-17.43, 15.12) |
|  | %NMF | -4.55 (-19.48, 10.37) | 0.06 (-15.01, 16.39) |
|  | NO2 | 0.54 ( 0.00, 0.95) | 0.22 ( 0.00, 0.74) |
|  | O3 | 0.14 ( 0.00, 0.66) | 0.25 ( 0.00, 0.80) |
|  | PM25 | 0.14 ( 0.00, 0.64) | 0.25 ( 0.00, 0.79) |
|  | SO2 | 0.17 ( 0.00, 0.57) | 0.28 ( 0.00, 0.80) |

Parameters were estimated using Dirichlet Bayesian weighted quantile sum (DBWQS) regression models with two independent chains, each with 30,000 iterations, including 15,000 burn-in iterations. Models were adjusted for maternal age, parity, education, household income, household size, marital status, race/ethnicity, country of origin, folic acid supplementation, healthy eating index, study site, and fetal sex.

# Abbreviations: 5MTHF, 5-methyltetrahydrofolate; UMFA, unmetabolized folic acid; NMF, non-methylated folate; NO2, nitrogen dioxide; O3, ozone; PM25, particulate matter 2.5; SO2, sulfur dioxide

# Table S6. Mean (95% Credible Interval) Posterior Estimates of Expected Differences (nmol/L) in Folate Vitamer Concentrations per 1-Quartile Difference in Air Pollution Mixture and Component Weights in the First and Third Trimesters among Pregnant Women in Canada, MIREC Study, Canada, 2008-2011

| Parameter | First Trimester | Third Trimester |
| --- | --- | --- |
| 5MTHF | 0.61 ( -2.06, 3.19) | 0.30 ( -2.61, 3.11) |
| NO2 | 0.26 ( 0.01, 0.70) | 0.25 ( 0.01, 0.68) |
| O3 | 0.24 ( 0.01, 0.66) | 0.26 ( 0.01, 0.69) |
| PM25 | 0.28 ( 0.01, 0.74) | 0.25 ( 0.01, 0.71) |
| SO2 | 0.22 ( 0.01, 0.68) | 0.25 ( 0.01, 0.70) |
| UMFA | -2.19 ( -6.83, 3.11) | 11.08 ( 2.20, 19.98) |
| NO2 | 0.33 ( 0.01, 0.76) | 0.13 ( 0.00, 0.41) |
| O3 | 0.19 ( 0.01, 0.64) | 0.45 ( 0.10, 0.76) |
| PM25 | 0.18 ( 0.00, 0.62) | 0.22 ( 0.01, 0.57) |
| SO2 | 0.30 ( 0.01, 0.72) | 0.20 ( 0.01, 0.49) |
| NMF | -0.42 ( -0.68, -0.17) | 1.02 ( 0.59, 1.48) |
| NO2 | 0.20 ( 0.01, 0.51) | 0.12 ( 0.00, 0.32) |
| O3 | 0.15 ( 0.01, 0.39) | 0.09 ( 0.00, 0.24) |
| PM25 | 0.53 ( 0.13, 0.85) | 0.12 ( 0.00, 0.33) |
| SO2 | 0.12 ( 0.00, 0.36) | 0.68 ( 0.45, 0.89) |

# Abbreviations: 5MTHF, 5-methyltetrahydrofolate; UMFA, unmetabolized folic acid; NMF, non-methylated folate; NO2, nitrogen dioxide; O3, ozone; PM25, particulate matter 2.5; SO2, sulfur dioxide

# Table S7. Mean (95% Credible Interval) Posterior Estimates of Expected Differences (nmol/L) in Plasma Total Folate and Folate Vitamers per 1-Quartile Difference in Air Pollution Mixture and Component Weights in the First and Third Trimesters among Pregnant Women in Canada by Fetal Sex, MIREC Study, Canada, 2008-2011

| Fetal Sex | Parameter | First Trimester | Third Trimester |
| --- | --- | --- | --- |
| Male | Total Folate | -2.12 (-10.94, 6.86) | 11.23 ( -3.27, 26.31) |
|  | NO2 | 0.26 ( 0.01, 0.68) | 0.18 ( 0.01, 0.56) |
|  | O3 | 0.29 ( 0.01, 0.72) | 0.29 ( 0.02, 0.67) |
|  | PM25 | 0.22 ( 0.01, 0.75) | 0.22 ( 0.01, 0.64) |
|  | SO2 | 0.23 ( 0.01, 0.66) | 0.32 ( 0.02, 0.70) |
|  | 5MTHF | -0.01 ( -4.02, 3.77) | 0.96 ( -2.74, 4.75) |
|  | NO2 | 0.24 ( 0.01, 0.68) | 0.24 ( 0.01, 0.68) |
|  | O3 | 0.26 ( 0.01, 0.70) | 0.25 ( 0.01, 0.68) |
|  | PM25 | 0.26 ( 0.01, 0.72) | 0.25 ( 0.01, 0.70) |
|  | SO2 | 0.24 ( 0.01, 0.69) | 0.25 ( 0.01, 0.68) |
|  | UMFA | -1.98 ( -8.75, 5.21) | 9.82 ( -2.23, 22.44) |
|  | NO2 | 0.29 ( 0.01, 0.71) | 0.17 ( 0.01, 0.55) |
|  | O3 | 0.28 ( 0.01, 0.69) | 0.32 ( 0.02, 0.69) |
|  | PM25 | 0.21 ( 0.00, 0.74) | 0.22 ( 0.01, 0.62) |
|  | SO2 | 0.22 ( 0.01, 0.64) | 0.30 ( 0.02, 0.68) |
|  | NMF | -0.39 ( -0.78, 0.02) | 1.27 ( 0.57, 1.95) |
|  | NO2 | 0.37 ( 0.03, 0.76) | 0.10 ( 0.00, 0.30) |
|  | O3 | 0.18 ( 0.01, 0.49) | 0.10 ( 0.00, 0.28) |
|  | PM25 | 0.31 ( 0.02, 0.74) | 0.10 ( 0.00, 0.31) |
|  | SO2 | 0.14 ( 0.00, 0.48) | 0.71 ( 0.43, 0.91) |

| Female | Total Folate | -5.09 (-14.04, 7.83) | 11.11 ( -5.71, 27.14) |
| --- | --- | --- | --- |
|  | NO2 | 0.22 ( 0.01, 0.62) | 0.20 ( 0.01, 0.60) |
|  | O3 | 0.17 ( 0.00, 0.70) | 0.37 ( 0.02, 0.76) |
|  | PM25 | 0.17 ( 0.01, 0.56) | 0.26 ( 0.01, 0.68) |
|  | SO2 | 0.45 ( 0.02, 0.85) | 0.17 ( 0.00, 0.57) |
|  | 5MTHF | 0.66 ( -2.89, 4.29) | -0.61 ( -5.39, 4.29) |
|  | NO2 | 0.25 ( 0.01, 0.69) | 0.23 ( 0.01, 0.66) |
|  | O3 | 0.27 ( 0.01, 0.71) | 0.24 ( 0.01, 0.67) |
|  | PM25 | 0.25 ( 0.01, 0.70) | 0.23 ( 0.01, 0.68) |
|  | SO2 | 0.23 ( 0.01, 0.71) | 0.31 ( 0.01, 0.78) |
|  | UMFA | -5.33 (-12.07, 5.07) | 10.66 ( -3.06, 23.68) |
|  | NO2 | 0.24 ( 0.01, 0.63) | 0.18 ( 0.01, 0.56) |
|  | O3 | 0.14 ( 0.00, 0.67) | 0.41 ( 0.03, 0.78) |
|  | PM25 | 0.15 ( 0.00, 0.51) | 0.24 ( 0.01, 0.64) |
|  | SO2 | 0.48 ( 0.02, 0.86) | 0.17 ( 0.01, 0.51) |
|  | NMF | -0.54 ( -0.88, -0.20) | 0.85 ( 0.29, 1.43) |
|  | NO2 | 0.15 ( 0.00, 0.46) | 0.28 ( 0.02, 0.63) |
|  | O3 | 0.17 ( 0.01, 0.43) | 0.11 ( 0.00, 0.32) |
|  | PM25 | 0.54 ( 0.14, 0.86) | 0.26 ( 0.01, 0.63) |
|  | SO2 | 0.14 ( 0.01, 0.41) | 0.35 ( 0.05, 0.67) |

# Abbreviations: 5MTHF, 5-methyltetrahydrofolate; UMFA, unmetabolized folic acid; NMF, non-methylated folate; NO2, nitrogen dioxide; O3, ozone; PM25, particulate matter 2.5; SO2, sulfur dioxide

# Table S8. Mean (95% Credible Interval) Posterior Estimates of Expected Differences (nmol/L) in Plasma Total Folate and Folate Vitamers per 1-Quartile Difference in Air Pollution Mixture and Component Weights in the First and Third Trimesters among Pregnant Women in Canada by Folic Acid Supplementation, MIREC Study, Canada, 2008-2011

| Folic Acid (µg/day) | Parameter | First Trimester | Third Trimester |
| --- | --- | --- | --- |
| <400 | 5MTHF | -5.25 (-16.74, 9.03) | 12.60 ( -3.06, 23.97) |
|  | NO2 | 0.18 ( 0.01, 0.60) | 0.56 ( 0.07, 0.88) |
|  | O3 | 0.41 ( 0.01, 0.82) | 0.13 ( 0.00, 0.44) |
|  | PM25 | 0.19 ( 0.01, 0.67) | 0.15 ( 0.00, 0.50) |
|  | SO2 | 0.22 ( 0.01, 0.62) | 0.17 ( 0.01, 0.51) |
|  | UMFA | 3.72 (-15.78, 21.38) | 12.68 (-22.05, 48.95) |
|  | NO2 | 0.27 ( 0.01, 0.73) | 0.21 ( 0.01, 0.65) |
|  | O3 | 0.23 ( 0.01, 0.73) | 0.29 ( 0.01, 0.71) |
|  | PM25 | 0.25 ( 0.01, 0.70) | 0.28 ( 0.01, 0.73) |
|  | SO2 | 0.24 ( 0.01, 0.67) | 0.23 ( 0.01, 0.66) |
|  | NMF | 0.08 ( -1.24, 1.38) | 3.78 ( 1.34, 6.22) |
|  | NO2 | 0.24 ( 0.01, 0.68) | 0.21 ( 0.01, 0.52) |
|  | O3 | 0.26 ( 0.01, 0.68) | 0.14 ( 0.01, 0.38) |
|  | PM25 | 0.26 ( 0.01, 0.70) | 0.16 ( 0.01, 0.49) |
|  | SO2 | 0.24 ( 0.01, 0.70) | 0.48 ( 0.12, 0.80) |
| 400-1000 | 5MTHF | 1.57 ( -1.22, 4.42) | 0.07 ( -3.64, 3.82) |
|  | NO2 | 0.31 ( 0.01, 0.74) | 0.24 ( 0.01, 0.68) |
|  | O3 | 0.23 ( 0.01, 0.62) | 0.26 ( 0.01, 0.70) |
|  | PM25 | 0.25 ( 0.01, 0.68) | 0.25 ( 0.01, 0.71) |
|  | SO2 | 0.21 ( 0.01, 0.62) | 0.26 ( 0.01, 0.72) |
|  | UMFA | -2.03 ( -7.17, 3.91) | 14.26 ( 3.41, 25.28) |
|  | NO2 | 0.30 ( 0.01, 0.73) | 0.12 ( 0.00, 0.39) |
|  | O3 | 0.20 ( 0.01, 0.70) | 0.34 ( 0.04, 0.65) |
|  | PM25 | 0.20 ( 0.01, 0.62) | 0.28 ( 0.02, 0.64) |
|  | SO2 | 0.31 ( 0.01, 0.75) | 0.26 ( 0.02, 0.58) |
|  | NMF | -0.41 ( -0.73, -0.09) | 0.90 ( 0.25, 1.53) |
|  | NO2 | 0.24 ( 0.01, 0.62) | 0.14 ( 0.01, 0.42) |
|  | O3 | 0.16 ( 0.01, 0.44) | 0.14 ( 0.01, 0.38) |
|  | PM25 | 0.50 ( 0.06, 0.85) | 0.14 ( 0.01, 0.44) |
|  | SO2 | 0.10 ( 0.00, 0.36) | 0.57 ( 0.20, 0.86) |
| >1000 | 5MTHF | -1.11 ( -7.02, 5.04) | 0.22 ( -5.40, 5.85) |
|  | NO2 | 0.22 ( 0.01, 0.67) | 0.25 ( 0.01, 0.68) |
|  | O3 | 0.24 ( 0.01, 0.66) | 0.25 ( 0.01, 0.68) |
|  | PM25 | 0.23 ( 0.01, 0.68) | 0.25 ( 0.01, 0.70) |
|  | SO2 | 0.30 ( 0.01, 0.76) | 0.26 ( 0.01, 0.69) |
|  | UMFA | -4.21 (-14.00, 5.40) | -5.16 (-24.40, 15.00) |
|  | NO2 | 0.28 ( 0.01, 0.70) | 0.24 ( 0.01, 0.67) |
|  | O3 | 0.24 ( 0.01, 0.66) | 0.22 ( 0.01, 0.71) |
|  | PM25 | 0.22 ( 0.01, 0.65) | 0.27 ( 0.01, 0.73) |
|  | SO2 | 0.26 ( 0.01, 0.67) | 0.27 ( 0.01, 0.70) |
|  | NMF | -0.51 ( -1.02, 0.00) | -0.29 ( -1.38, 0.94) |
|  | NO2 | 0.24 ( 0.01, 0.62) | 0.21 ( 0.01, 0.64) |
|  | O3 | 0.15 ( 0.01, 0.49) | 0.35 ( 0.01, 0.80) |
|  | PM25 | 0.34 ( 0.02, 0.75) | 0.22 ( 0.01, 0.64) |
|  | SO2 | 0.26 ( 0.01, 0.65) | 0.22 ( 0.01, 0.74) |

# Abbreviations: 5MTHF, 5-methyltetrahydrofolate; UMFA, unmetabolized folic acid; NMF, non-methylated folate; NO2, nitrogen dioxide; O3, ozone; PM25, particulate matter 2.5; SO2, sulfur dioxide

# Figure S1. Plasma Total Folate by Diet Quality Index, Stratified by Folic Acid Supplementation, in the First and Third Trimesters, MIREC, Canada, 2008-2011


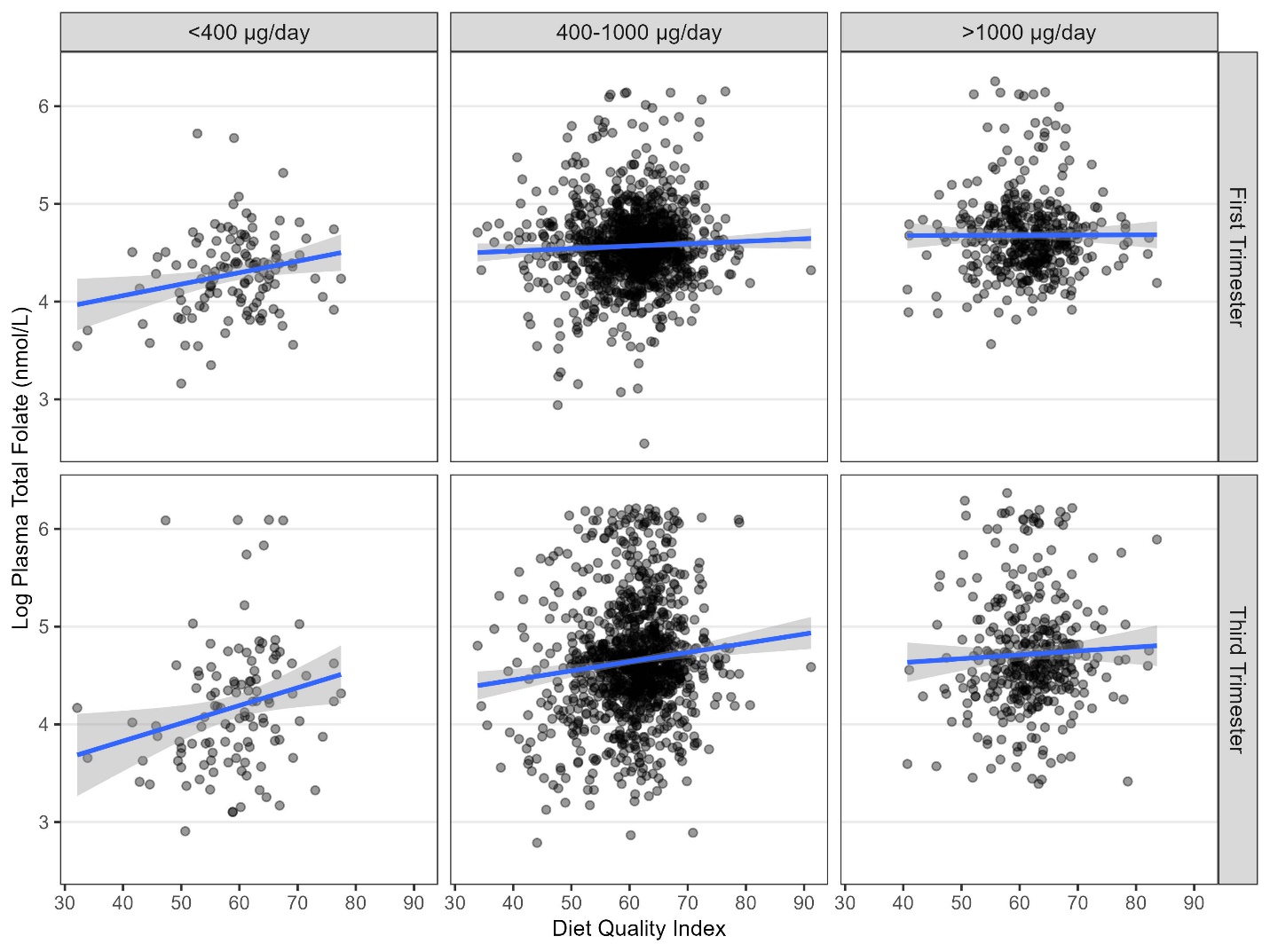


Figures include data from all MIREC participants with non-missing folic acid supplementation, diet quality index, and plasma total folate in the specified trimester. The numbers of participants in each panel are 122 and 113 for <400 µg/day, 1,147 and 1,073 for 400-1,000 µg/day, and 412 and 369 for >1,000 µg/day in the first and third trimesters, respectively.

# Figure S2. Inclusion of Participants in Analysis of Air Pollution Mixture and Plasma Folate among Pregnant Women, MIREC, Canada, 2008-2011

**
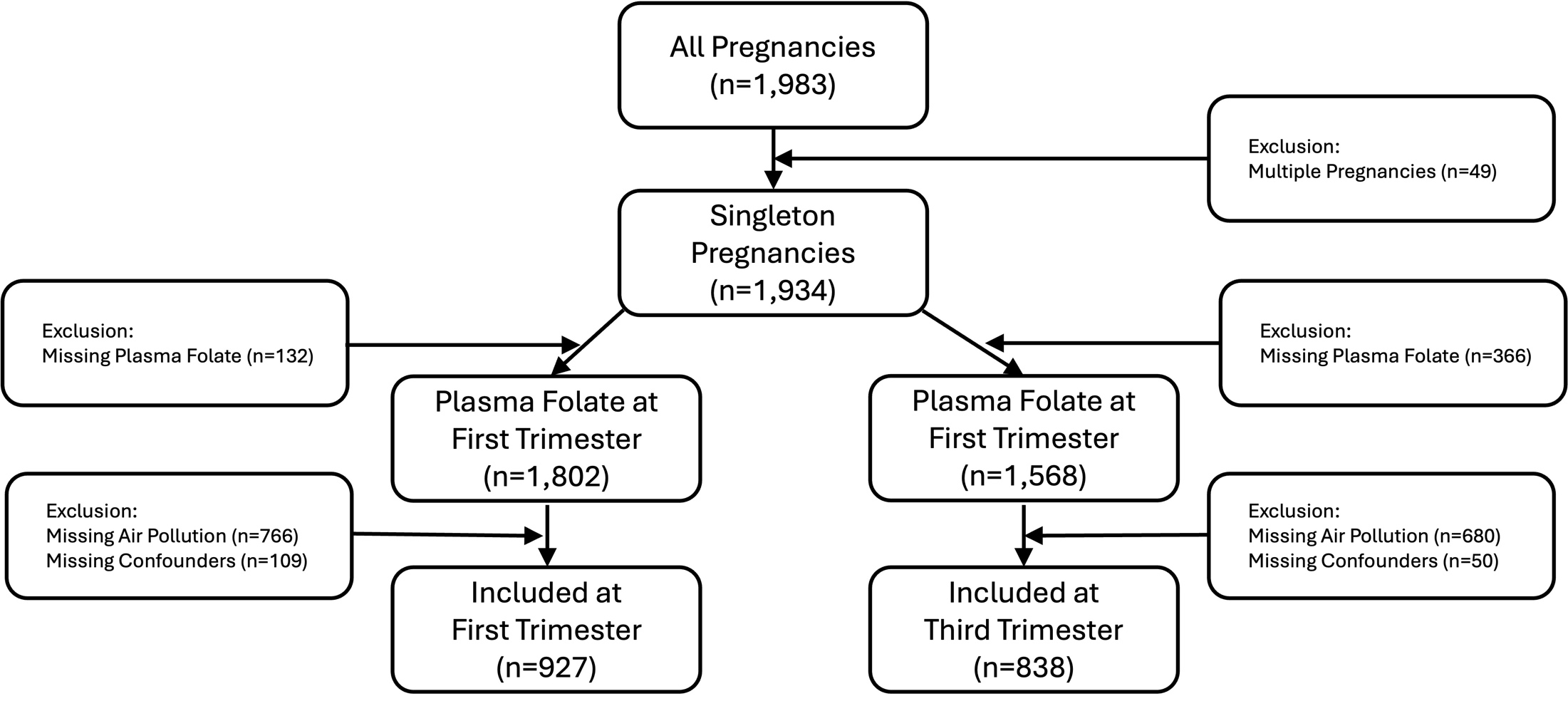
**

# Figure S3. Mean (95% Credible Interval) Posterior Estimates of Expected Differences and Component Weights for Air Pollution and Plasma 5MTHF Concentrations in the First and Third Trimesters among Pregnant Women in Canada, MIREC Study, Canada, 2008-2011


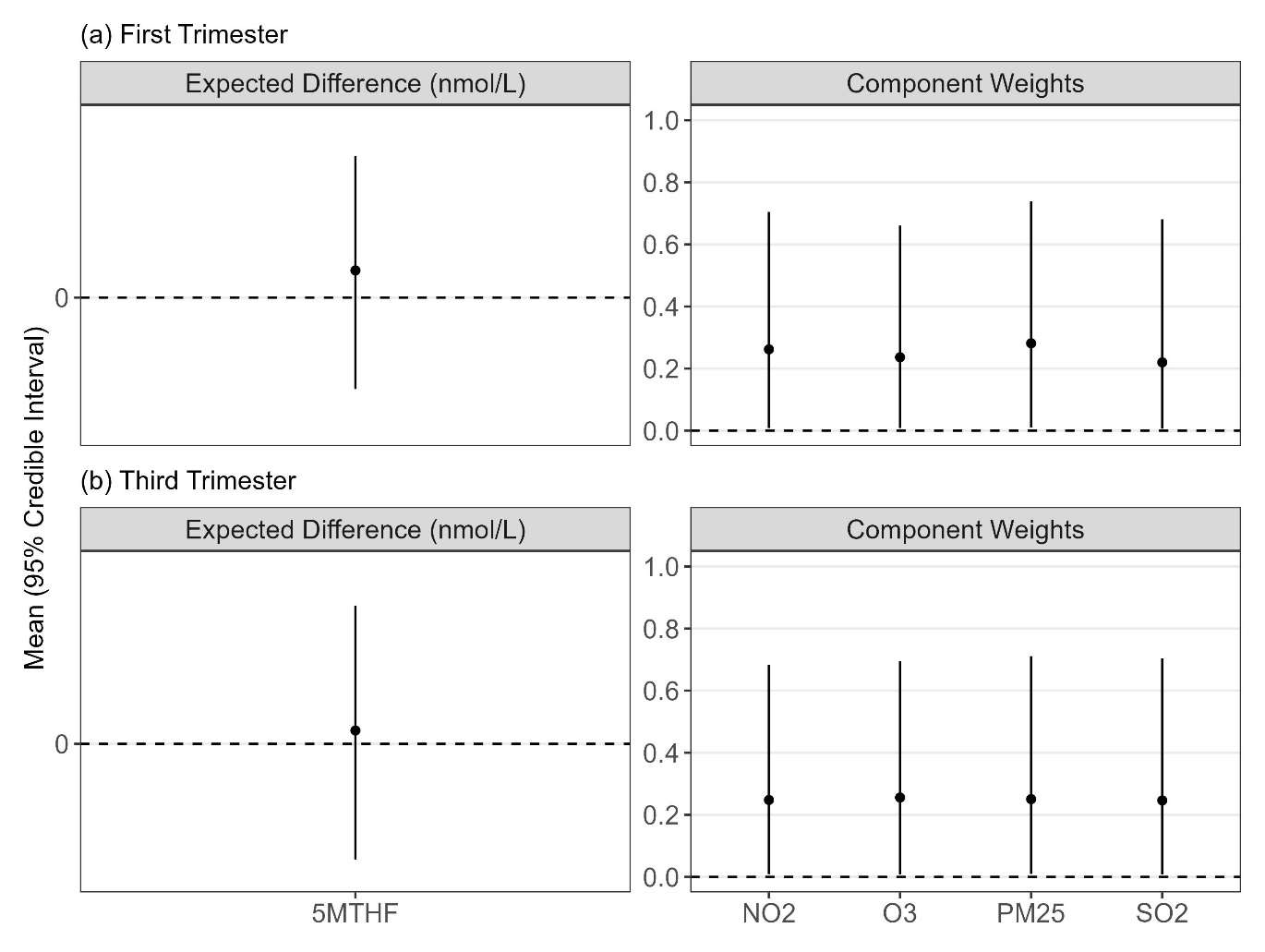


# Abbreviations: 5MTHF, 5-methyltetrahydrofolate; NO2, nitrogen dioxide; O3, ozone; PM25, particulate matter 2.5; SO2, sulfur dioxide

# Figure S4. Mean (95% Credible Interval) Posterior Estimates of Expected Differences and Component Weights for Air Pollution and Plasma UMFA Concentrations in the First and Third Trimesters among Pregnant Women in Canada, MIREC Study, Canada, 2008-2011


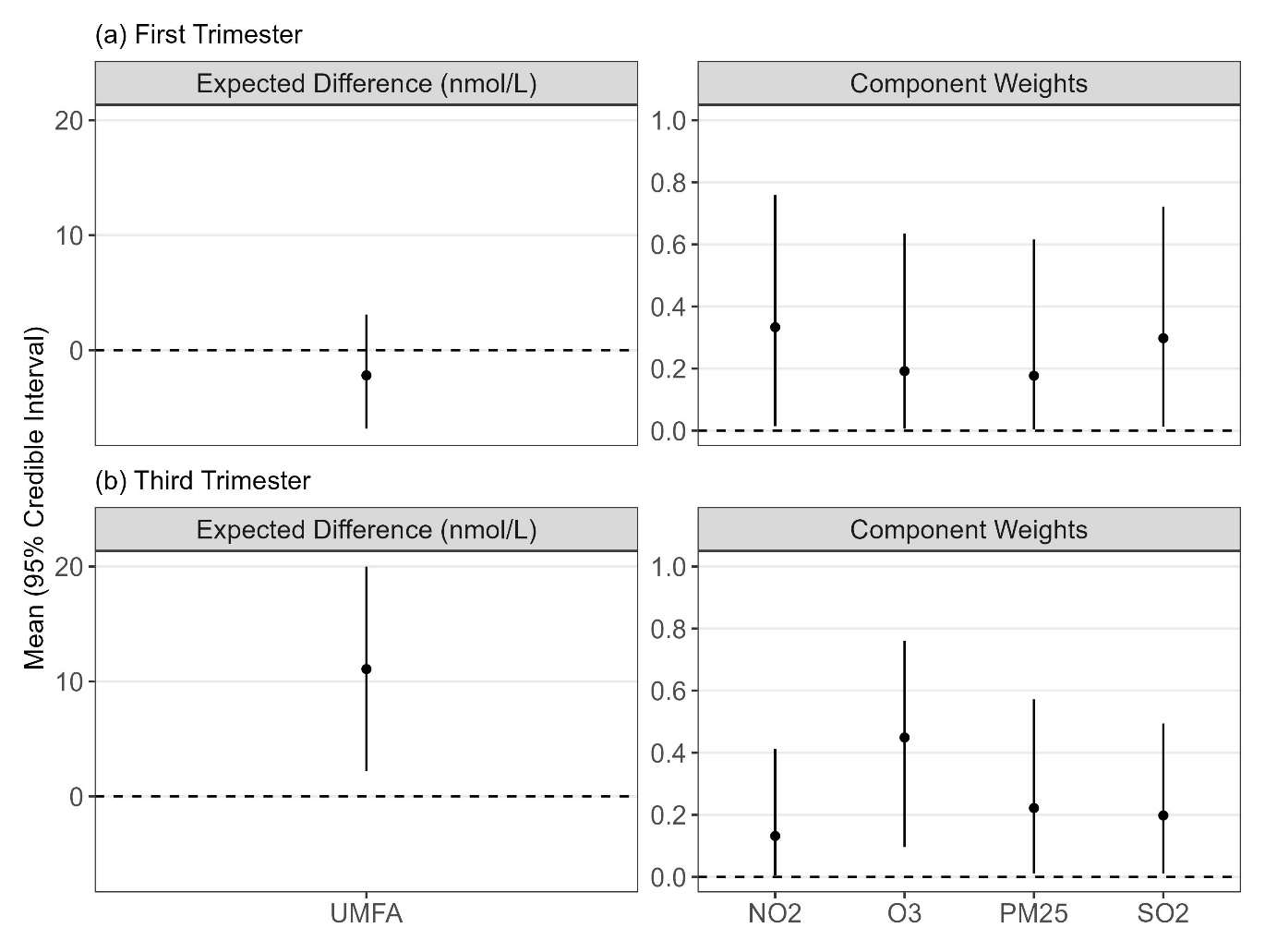


# Abbreviations: UMFA, unmetabolized folic acid; NO2, nitrogen dioxide; O3, ozone; PM25, particulate matter 2.5; SO2, sulfur dioxide

# Figure S5. Mean (95% Credible Interval) Posterior Estimates of Expected Differences and Component Weights for Air Pollution and Plasma NMF Concentrations in the First and Third Trimesters among Pregnant Women in Canada, MIREC Study, Canada, 2008-2011


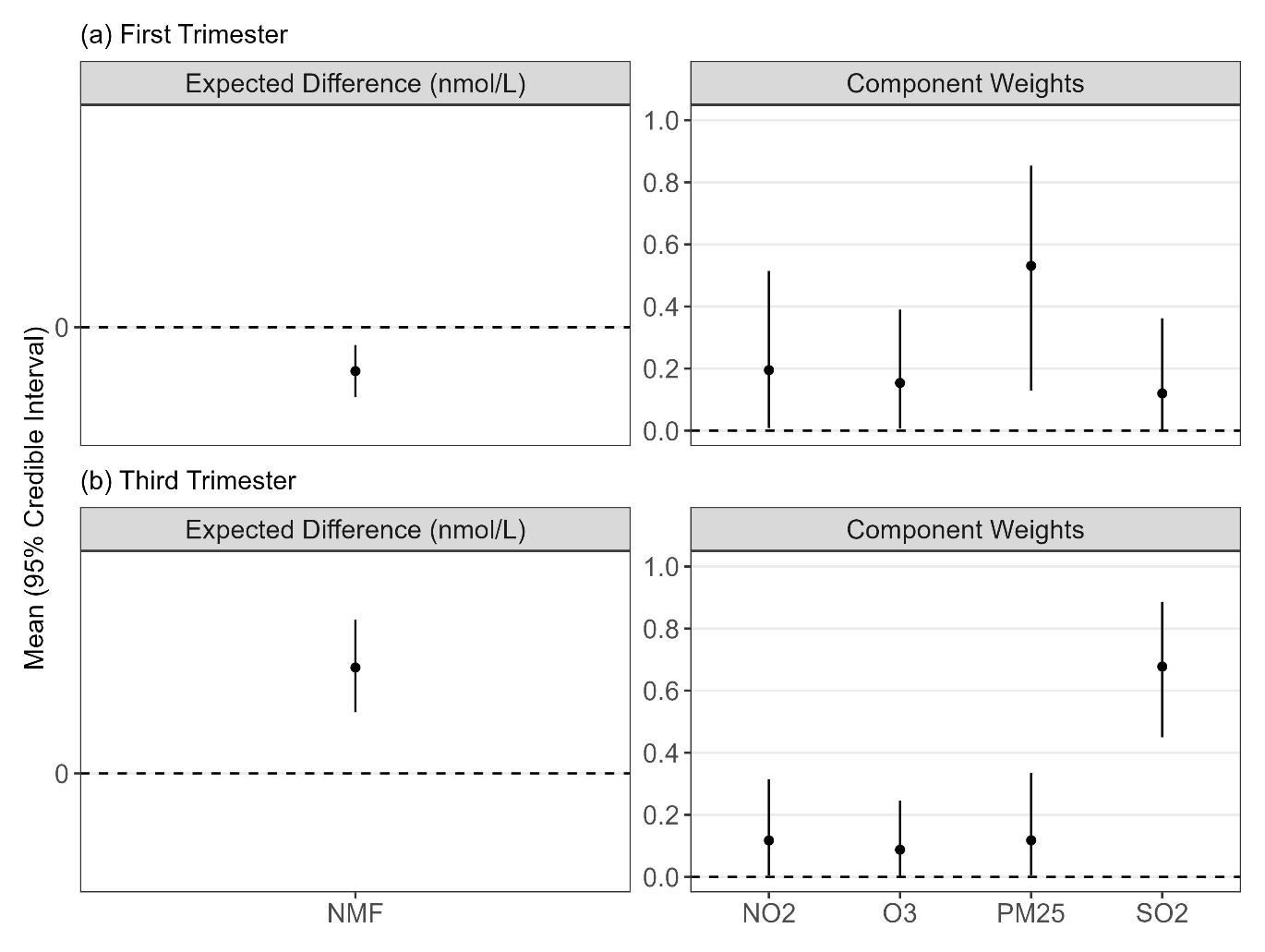


# Abbreviations: NMF, non-methylated folate; NO2, nitrogen dioxide; O3, ozone; PM25, particulate matter 2.5; SO2, sulfur dioxide

# Figure S6. Mean (95% Credible Interval) Posterior Estimates of Expected Differences and Component Weights for Air Pollution and Plasma 5MTHF Concentrations in the First and Third Trimesters among Pregnant Women by Fetal Sex in Canada, MIREC Study, Canada, 2008-2011


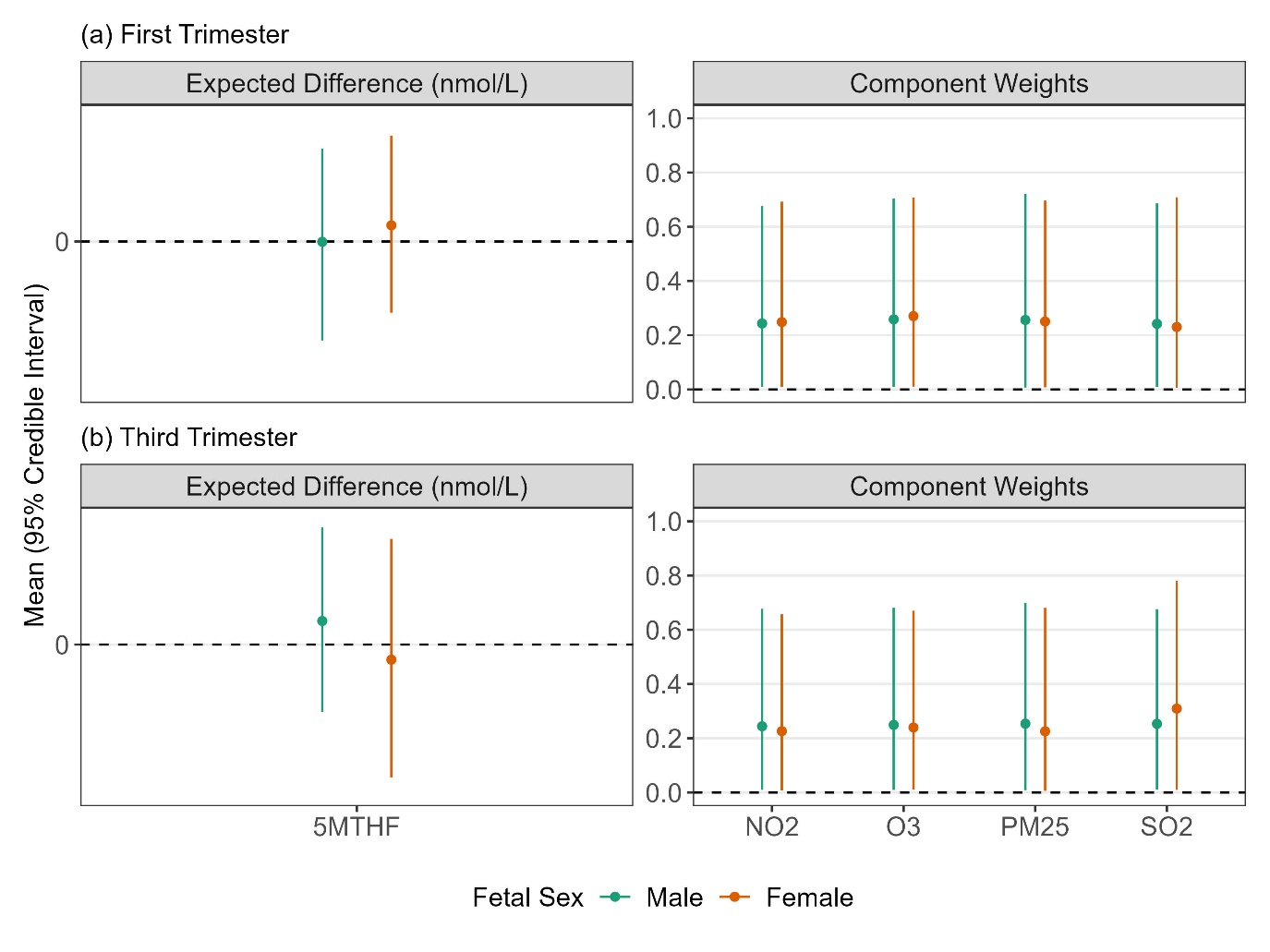


# Abbreviations: 5MTHF, 5-methyltetrahydrofolate; NO2, nitrogen dioxide; O3, ozone; PM25, particulate matter 2.5; SO2, sulfur dioxide

# Figure S7. Mean (95% Credible Interval) Posterior Estimates of Expected Differences and Component Weights for Air Pollution and Plasma UMFA Concentrations in the First and Third Trimesters among Pregnant Women by Fetal Sex in Canada, MIREC Study, Canada, 2008-2011


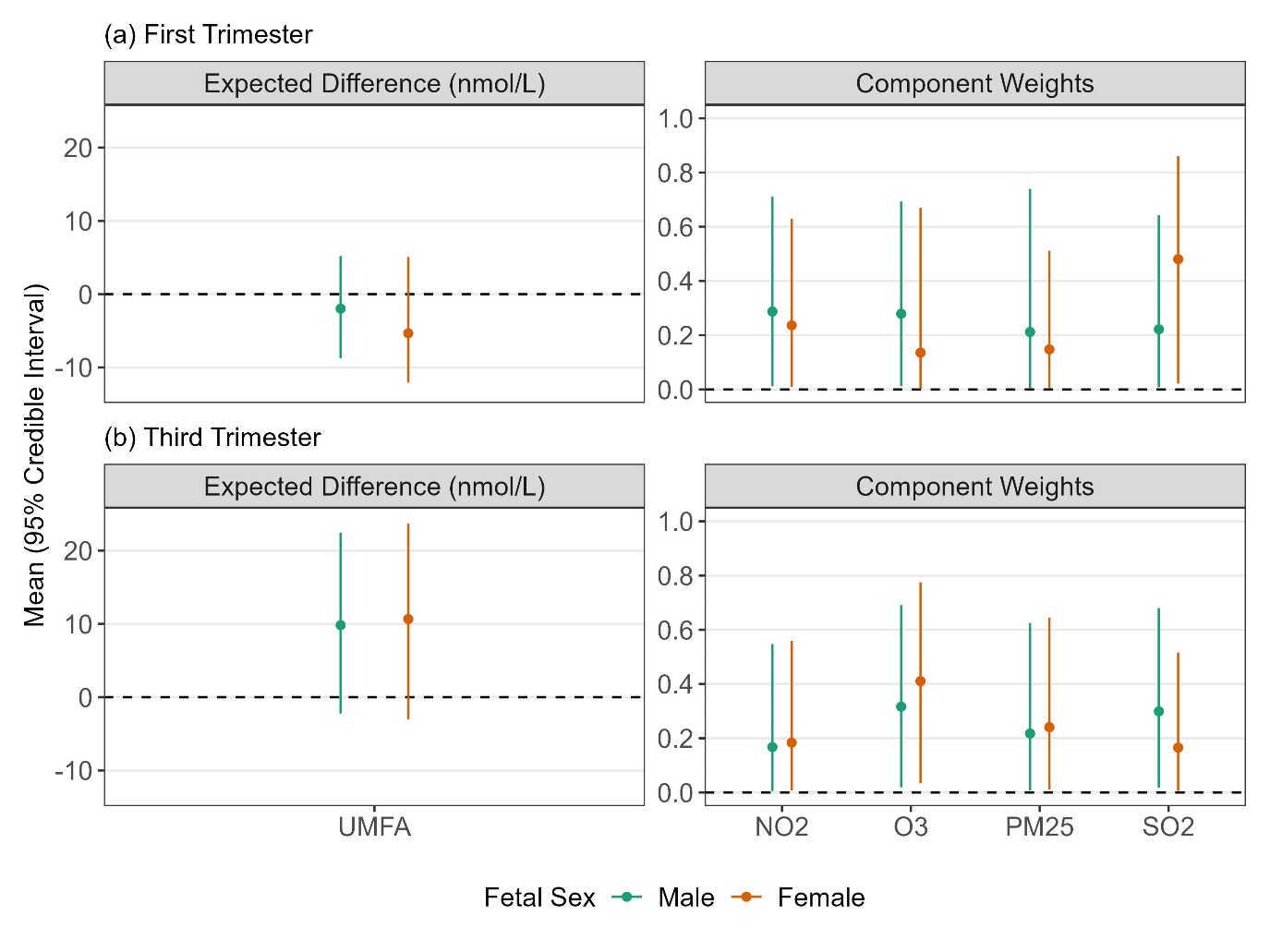


# Abbreviations: UMFA, unmetabolized folic acid; NO2, nitrogen dioxide; O3, ozone; PM25, particulate matter 2.5; SO2, sulfur dioxide

# Figure S8. Mean (95% Credible Interval) Posterior Estimates of Expected Differences and Component Weights for Air Pollution and Plasma NMF Concentrations in the First and Third Trimesters among Pregnant Women by Fetal Sex in Canada, MIREC Study, Canada, 2008-2011


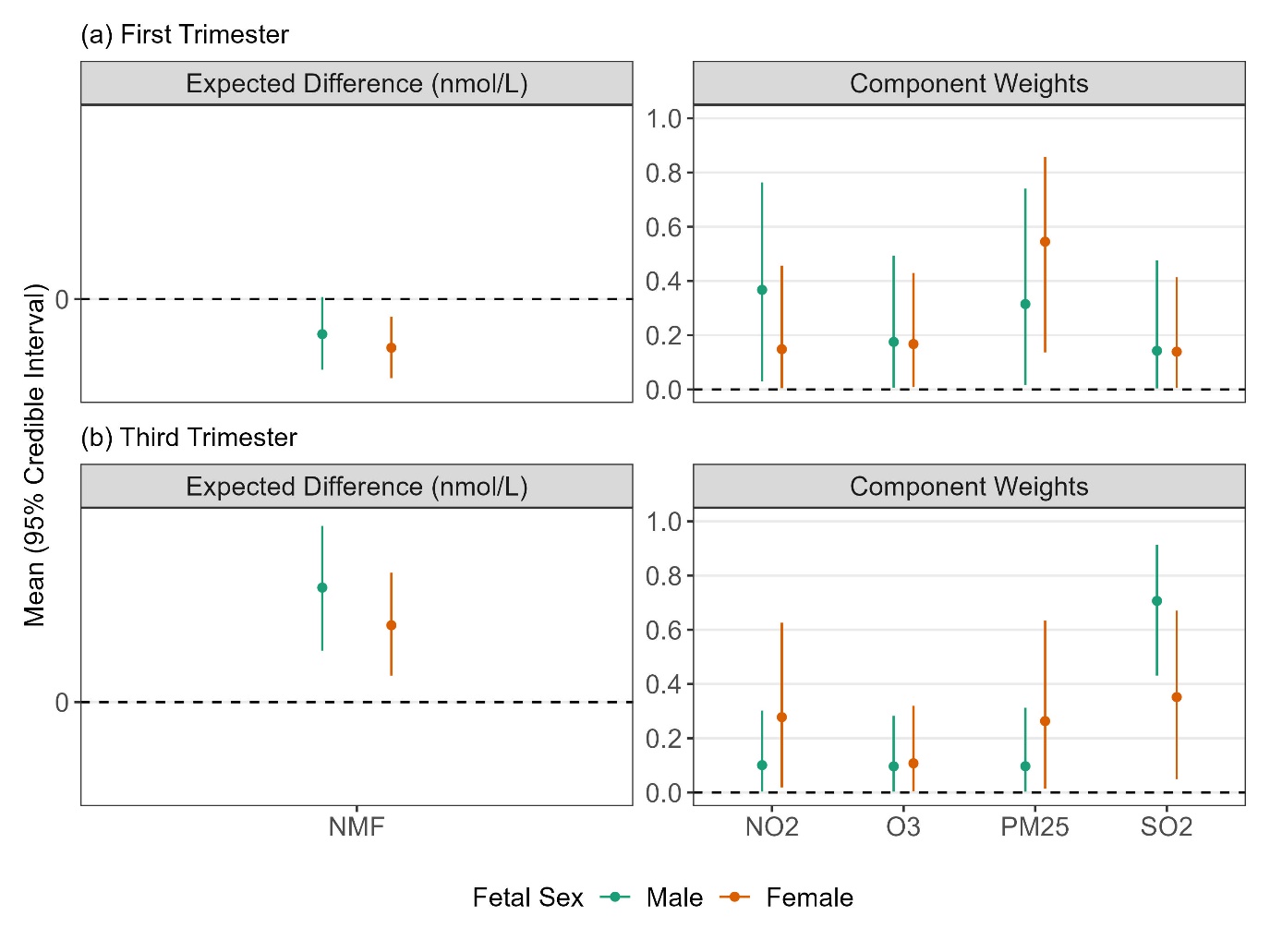


# Abbreviations: NMF, non-methylated folate; NO2, nitrogen dioxide; O3, ozone; PM25, particulate matter 2.5; SO2, sulfur dioxide

# Figure S9. Mean (95% Credible Interval) Posterior Estimates of Mixture Associations and Component Weights for Air Pollution and Plasma 5MTHF Concentrations in the First and Third Trimesters among Pregnant Women by Folic Acid Supplementation in Canada, MIREC Study, Canada, 2008-2011


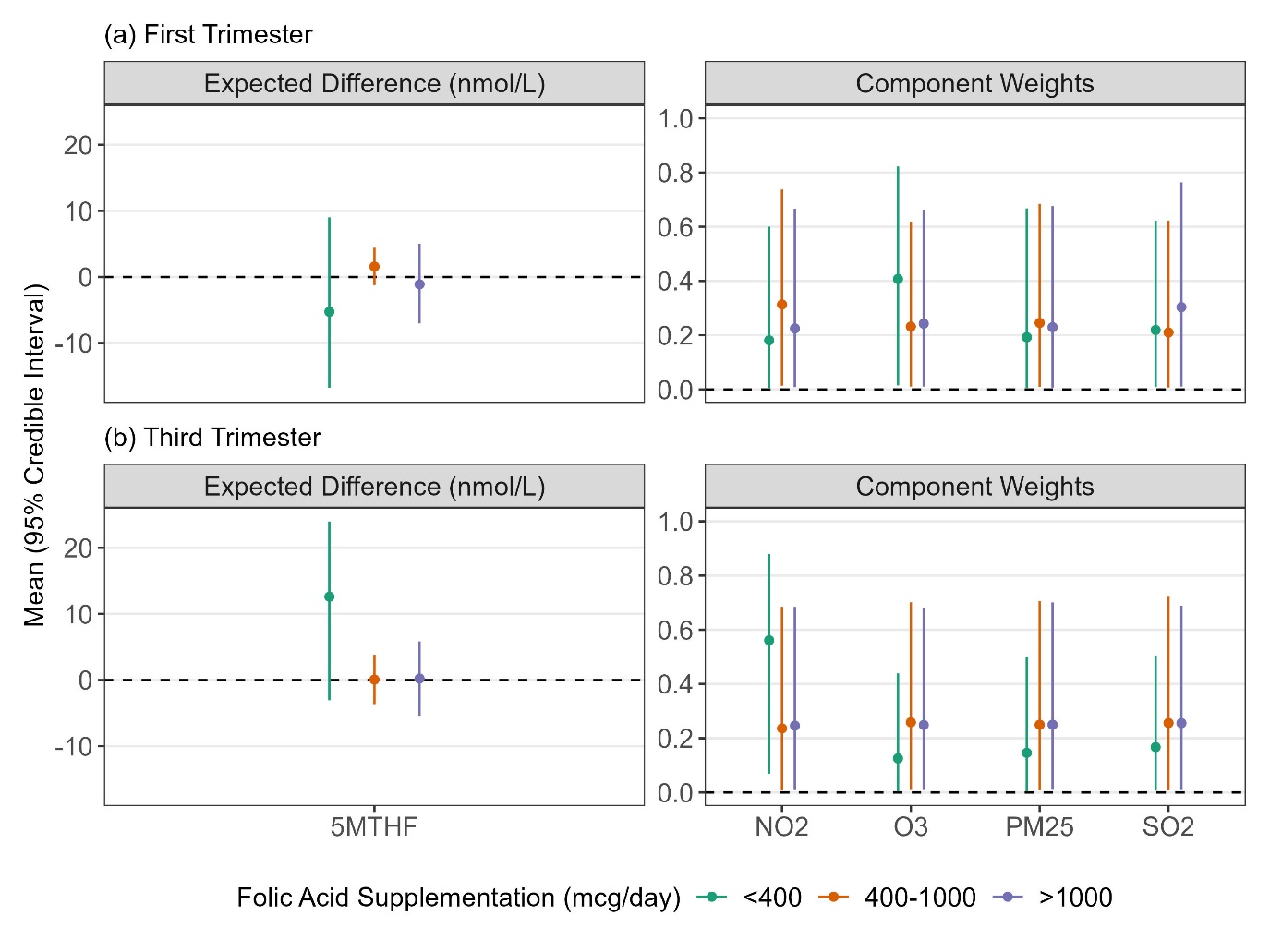


# Abbreviations: 5MTHF, 5-methyltetrahydrofolate; NO2, nitrogen dioxide; O3, ozone; PM25, particulate matter 2.5; SO2, sulfur dioxide

# Figure S10. Mean (95% Credible Interval) Posterior Estimates of Mixture Associations and Component Weights for Air Pollution and Plasma UMFA Concentrations in the First and Third Trimesters among Pregnant Women by Folic Acid Supplementation in Canada, MIREC Study, Canada, 2008-2011


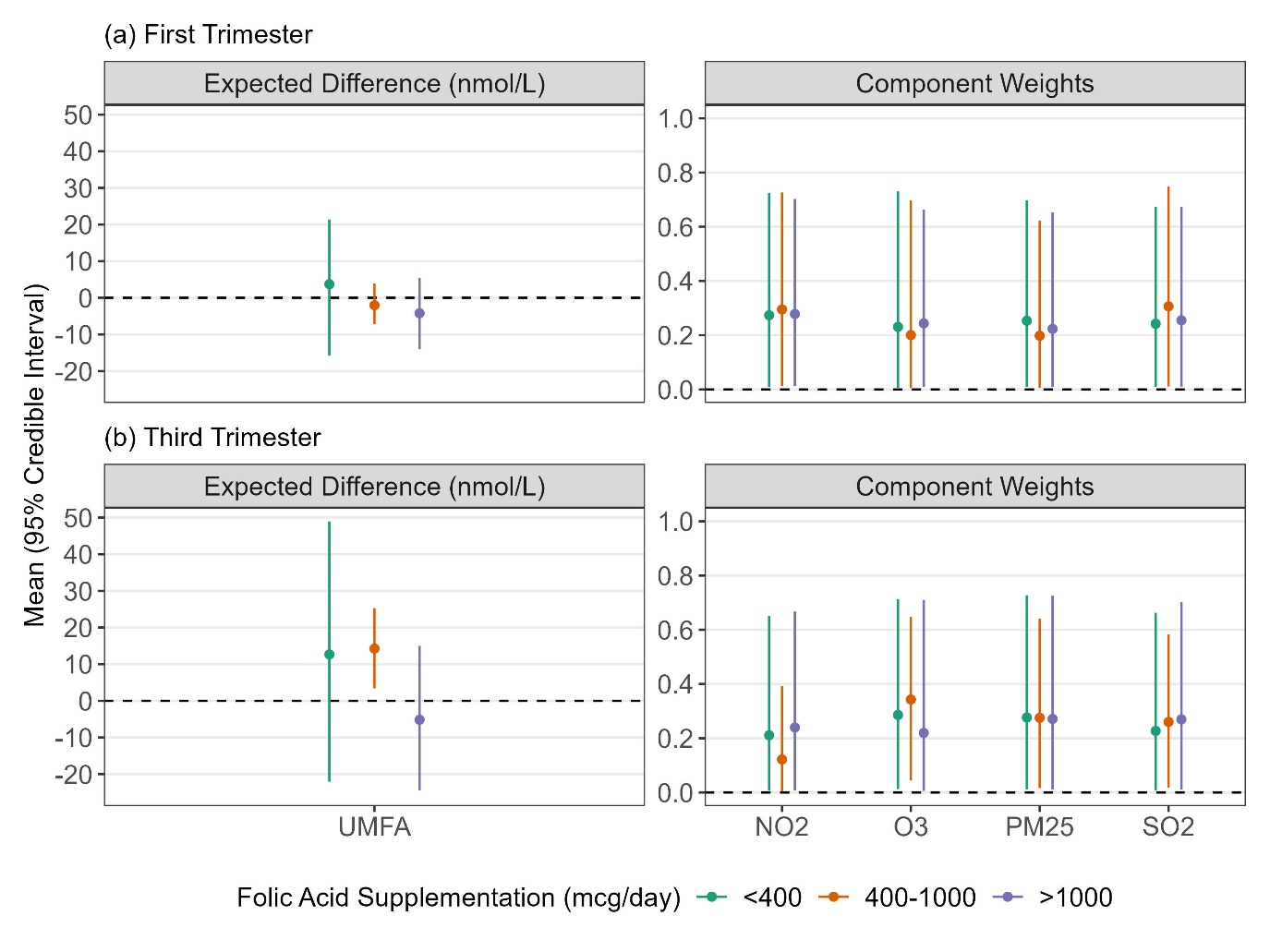


# Abbreviations: UMFA, unmetabolized folic acid; NO2, nitrogen dioxide; O3, ozone; PM25, particulate matter 2.5; SO2, sulfur dioxide

# Figure S11. Mean (95% Credible Interval) Posterior Estimates of Mixture Associations and Component Weights for Air Pollution and Plasma NMF Concentrations in the First and Third Trimesters among Pregnant Women by Folic Acid Supplementation in Canada, MIREC Study, Canada, 2008-2011


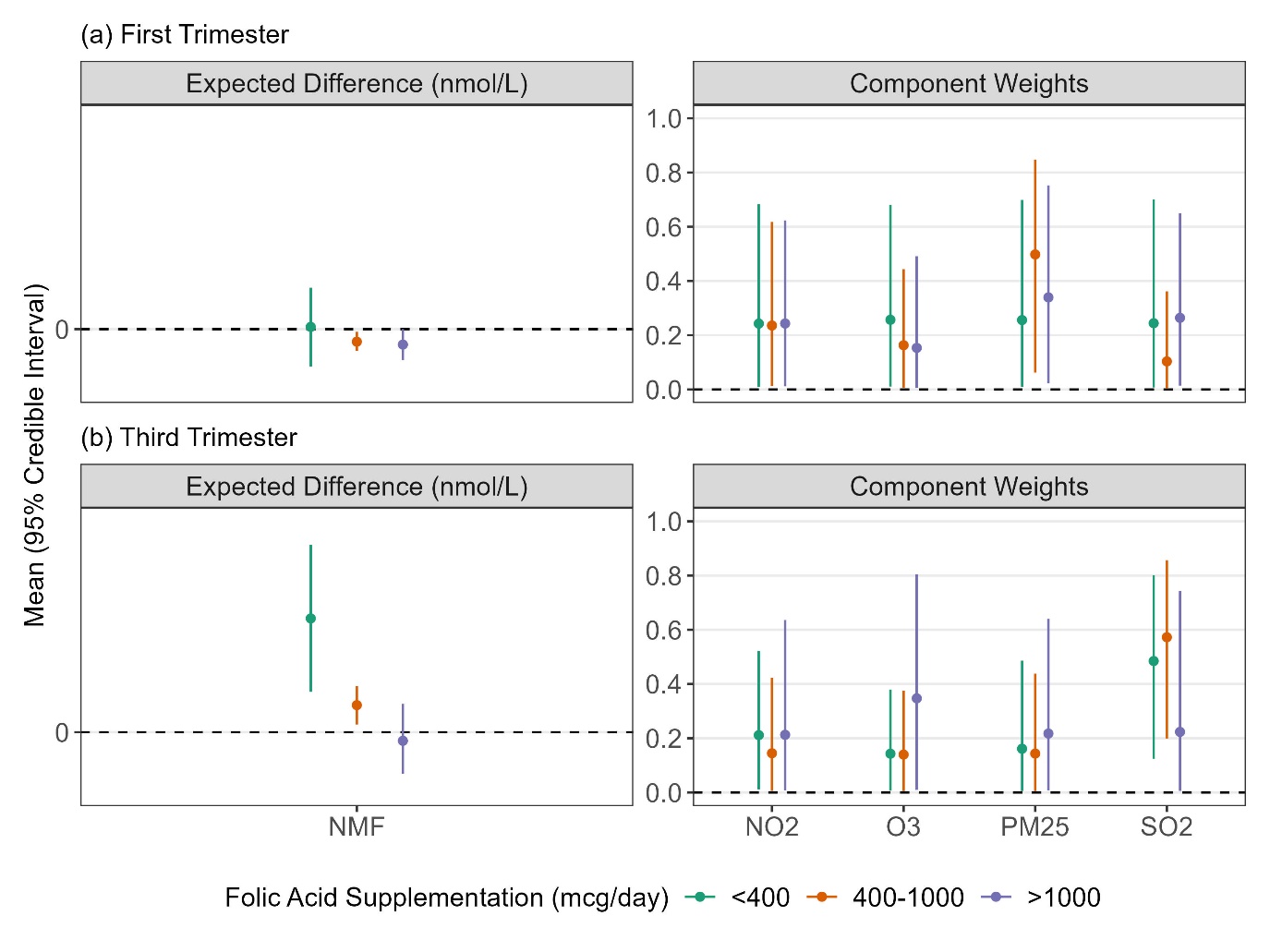


# Abbreviations: NMF, non-methylated folate; NO2, nitrogen dioxide; O3, ozone; PM25, particulate matter 2.5; SO2, sulfur dioxide
